# The U.S. COVID-19 County Policy Database: a novel resource to support pandemic-related research

**DOI:** 10.1186/s12889-022-14132-6

**Published:** 2022-10-10

**Authors:** Rita Hamad, Kristin A. Lyman, Feng Lin, Madelaine F. Modrow, Pelin Ozluk, Kristen M. J. Azar, Amie Goodin, Carmen R. Isasi, Heather E. Kitzman, Sara J. Knight, Gregory M. Marcus, Cheryl N. McMahill-Walraven, Paul Meissner, Vinit Nair, Emily C. O’Brien, Jeffrey E. Olgin, Noah D. Peyser, Gosia Sylwestrzak, Natasha Williams, Mark J. Pletcher, Thomas Carton

**Affiliations:** 1 Department of Family and Community Medicine, Philip R. Lee Institute for Health Policy Studies, University of California San Francisco, 995 Potrero Avenue, Building 80, Ward 83, San Francisco, CA 94110 USA; 2 Louisiana Public Health Institute, New Orleans, Louisiana USA; 3 Department of Epidemiology and Biostatistics, University of California San Francisco, San Francisco, CA USA; 4 Enterprise Health Services Research, Anthem, Inc, Wilmington, Delaware USA; 5 Institute for Advancing Health Equity, Sutter Health, Sacramento, CA USA; 6 Pharmaceutical Outcomes and Policy, Center for Drug Evaluation and Safety, University of Florida, Gainesville, FL USA; 7 Department of Epidemiology and Population Health, Albert Einstein College of Medicine, Bronx, NY USA; 8 Baylor Scott and White Health, Dallas, TX USA; 9 Robbins Institute for Health Policy and Leadership, Baylor University, Waco, TX USA; 10 Department of Internal Medicine, Division of Epidemiology, University of Utah, Salt Lake City, Utah USA; 11 Division of Cardiology, University of California San Francisco, San Francisco, CA USA; 12 Clinical Trial Services, CVS Health, Blue Bell, Pennsylvania USA; 13 Montefiore Medical Center , Albert Einstein College of Medicine, Bronx, NY USA; 14 Practice Research Network, Humana Healthcare Research, Sharon, MA USA; 15 Duke Clinical Research Institute, Duke University, Durham, NC USA; 16 New York University Grossman School of Medicine, New York City, NY USA

**Keywords:** COVID-19 pandemic, Policy evaluation, Economic support, Health policy

## Abstract

**Background:**

It is increasingly recognized that policies have played a role in both alleviating and exacerbating the health and economic consequences of the COVID-19 pandemic. There has been limited systematic evaluation of variation in U.S. local COVID-19-related policies. This study introduces the U.S. COVID-19 County Policy (UCCP) Database, whose objective is to systematically gather, characterize, and assess variation in U.S. county-level COVID-19-related policies.

**Methods:**

In January-March 2021, we collected an initial wave of cross-sectional data from government and media websites for 171 counties in 7 states on 22 county-level COVID-19-related policies within 3 policy domains that are likely to affect health: (1) containment/closure, (2) economic support, and (3) public health. We characterized the presence and comprehensiveness of policies using univariate analyses. We also examined the correlation of policies with one another using bivariate Spearman’s correlations. Finally, we examined geographical variation in policies across and within states.

**Results:**

There was substantial variation in the presence and comprehensiveness of county policies during January-March 2021. For containment and closure policies, the percent of counties with no restrictions ranged from 0% (for public events) to more than half for public transportation (67.8%), hair salons (52.6%), and religious gatherings (52.0%). For economic policies, 76.6% of counties had housing support, while 64.9% had utility relief. For public health policies, most were comprehensive, with 70.8% of counties having coordinated public information campaigns, and 66.7% requiring masks outside the home at all times. Correlations between containment and closure policies tended to be positive and moderate (i.e., coefficients 0.4–0.59). There was variation within and across states in the number and comprehensiveness of policies.

**Conclusions:**

This study introduces the UCCP Database, presenting granular data on local governments’ responses to the COVID-19 pandemic. We documented substantial variation within and across states on a wide range of policies at a single point in time. By making these data publicly available, this study supports future research that can leverage this database to examine how policies contributed to and continue to influence pandemic-related health and socioeconomic outcomes and disparities. The UCCP database is available online and will include additional time points for 2020–2021 and additional counties nationwide.

**Supplementary Information:**

The online version contains supplementary material available at 10.1186/s12889-022-14132-6.

## Introduction

The COVID-19 pandemic has resulted in over 1 million deaths in the U.S. as of May 2022 [1]. It has also caused tremendous financial hardship with nearly 15% unemployment at its peak in April 2020 [2], and millions falling into poverty [3]. It is increasingly recognized that health and social policies played a role in both alleviating and exacerbating the health and economic consequences of the pandemic. For example, mandated business closures likely contributed to decreased levels of transmission [4, 5], but also job and income loss that disproportionately affected low-income groups and women [6]. Meanwhile, economic policies like eviction moratoria ensured that families had the resources to stay healthy at home [7].

With limited coordination of federal guidance in COVID-19 policymaking relative to other high-income countries [8, 9], there has been substantial variation in state- and county-level COVID-19-related policies. The COVID-19 U.S. State Policy database at Boston University has systematically documented longitudinal variation in state policies on closures, shelter-in-place orders, housing protections, and more [10]. Similarly, the Oxford COVID-19 Government Response Tracker documents national-level policies over time and collects subnational data for countries with substantial local variation (including the U.S.) [11]. This database has documented substantial state-level variation in the comprehensiveness of containment and closure policies. Researchers have subsequently examined state policies’ effects on a variety of health and related outcomes, including whether minimum wage and paid sick leave policies affected food insufficiency during the pandemic [12].

In contrast, there has been little systematic documentation of *county*-level policies in the U.S., despite the fact that counties have been deeply involved in COVID-19 policymaking since early in the pandemic and that there are substantial differences in county-level COVID-19 rates even within a given state [13]. For example, six counties in the San Francisco Bay Area issued the first coordinated shelter-in-place orders in the U.S. on March 16, 2020 even before an analogous state policy was implemented [14]. A few studies have undertaken assessments of county policies for a handful of counties or for single type of policy (e.g., mask requirements) [15, 16]. Other studies seeking to explain differences in COVID-19-related outcomes have focused on county demographic characteristics such as population density or racial/ethnic composition, despite acknowledging the importance of policymaking [17, 18]. Without systematic and comprehensive documentation of county-level policies, it will be difficult to evaluate policies’ effects on health and economic outcomes.

In this study, we sought to fill this gap by gathering data on a range of county-level COVID-19-related policies from 171 counties in 7 states as the first phase of the new U.S. COVID-19 County Policy (UCCP) Database. We then characterize these policies both within and across states, testing the hypothesis that there is geographic variation in both the number and stringency of policies passed at the county and state levels.

## Methods

### Overview of data collection

The objective of the U.S. COVID-19 County Policy (UCCP) Database is to systematically gather, characterize, and assess variation in U.S. county-level COVID-19-related policies. This study summarizes the first phase of data collection, in which the research team gathered policy data for 171 counties in California, Louisiana, Mississippi, New Jersey, New York, Texas, and Utah (Supplemental Table 1). The total population of these counties is 90.4 million. While these counties are not nationally representative, they include over a quarter of the U.S. population and are diverse with respect to geography, race/ethnicity, and politics [19]. The reasons for the selection of the given counties is described in the Supplemental Methods.

For these counties, we gathered data in January-March 2021 on COVID-19-related policies that were in effect at that time, capturing a cross-sectional picture of county policies during that period. Data collection proceeded for approximately eight weeks. Data were also collected for the corresponding states in which these counties are nested, with two waves of state policy data collection conducted in January 2021 just before county data collection, and again in February–March 2021 just after county data collection was completed.

### Policy coding

We gathered data on 22 policies within three overarching domains: containment and closure, economic support, and public health measures (Table 1). These were in part modeled on national and state policy data currently collected through the Oxford COVID-19 Government Response Tracker, excluding those not applicable to counties (e.g., border control), and including additional policies that are primarily relevant at the county level that may affect health (e.g., housing support). For each policy, the study team assessed a sample of current policies across rural and urban counties in several states, and developed scoring criteria to assess comprehensiveness of the policy. These were aligned with the way in which policies and associated restrictions were framed at the county level (Supplemental Table 2). For example, one policy indicator captured public events with the following categories: minimal (≥ 50% capacity) limitations, major (< 50% capacity) limitations, recommended cancellation, and required cancellation. Each indicator also included a category to capture the scenario where there was explicitly no relevant restriction or program in place, and a category for missing where there was no information available to determine the policy/program in place. For school closures in this first phase of data collection, in counties with more than one school district or university, data collectors coded the district or university with the most stringent/comprehensive policy.

**Table 1:** County policies, by policy domains

Policy	Description
*Panel A. Containment and Closure Domain*	
Workplaces	Extent of closure of non-essential office work
Public events	Any event open to the public or through purchase of a ticket
Private gatherings	Any gathering not open to the public or through purchase of a ticket (e.g., weddings)
Public transport	Extent of closure and based on the most comprehensive policy
Stay at home orders	Presence/absence and associated exceptions (e.g., essential trips)
Gyms	Capacity restrictions
Restaurants	Indoor capacity restrictions, outdoor, and takeout/delivery
Bars	Indoor capacity restrictions, outdoor, and takeout/delivery
Movie theaters	Capacity restrictions
Schools	Extent of closure of public schools and universities
Childcare settings	Scoring aligned with school closing policy
Salons/barber shops	Capacity restrictions
Religious gatherings	Capacity restrictions
Nursing homes	Visitation limitations or ban
Curfews	Presence/absence, including requirements that bars close by a specific time
*Panel B. Economic Support Domain*	
Housing support	Presence/absence of program (e.g., rent payment support/relief, mortgage payment support/relief, eviction freezes/limitations)
Utility support	Presence/absence of program (e.g., flexible payments, utility bill discounts, utility shut-off freezes)
*Panel C. Public Health Domain*	
Public information	Level of comprehensiveness of campaign
Testing policy	Groups for which public testing is available
Contact tracing	Level of comprehensiveness of contact tracing efforts
Facial coverings	Extent of mask requirement in shared/public spaces
Vaccine availability	Vaccine availability by group (e.g., essential workers, elderly)

Additional details on scoring criteria for the policies included in the study are presented in Supplemental Table 2

### Data collection

Data collectors used the Research Electronic Data Capture (REDCap) data entry and management platform [20]. For each policy, data collectors abstracted scoring related to comprehensiveness, the effective date of the policy in place at the time of data collection, and source documentation. Data collectors gathered data from a variety of sources, including government websites, policy and government response summaries and databases, press releases, news articles, and social media posts by government organizations. Details on data collection are provided in the Supplemental Methods.

### Imputation of missing data

Despite a thorough search of multiple sources, in some cases there was no information on the county policies of interest. This ranged from 4.7% for school closures to 50.3% for utility support (Supplemental Table 3). This was especially the case in rural counties (39.9% missingness across all policies), which are less likely to have robust public health departments than urban counties (23.8% missing) (Supplemental Table 4). For these, data on state policies were used to impute county policies. Since state data were gathered in two waves—just before and after county data collection—we used the closest state survey date to fill in the corresponding policy for missing county data. If there was no information on the policy at the state level either, then the policy remained coded as missing.

Additionally, we combined policies that were missing and those with clear documentation of no relevant restrictions, in essence assuming that there was no policy in place for those counties with no documented policy. While this assumption may not always be accurate, if policies were difficult for our trained staff to find after a thorough search, they were also likely to be difficult for residents to locate, resulting in no substantive restrictions in place. For example, counties with no clear documentation of a face covering policy and those with clear documentation that no face coverings were required were combined.

### Primary analysis

First, we calculated univariate distributions of each policy within each of the three overarching policy domains, documenting the range of comprehensiveness of each policy.

We then calculated bivariate Spearman’s correlations between each pair of policies. This type of non-parametric analysis examines the extent to which two ordinal ranked variables are associated with one another. In this case, it assessed the extent to which policies co-occurred in a given county, reflecting the fact that governments often implement bundles of policies on related issues [21, 22].

Next, we examined geographic distribution of the policies. First, for each state, we tabulated the mean number of policies implemented by counties in that state in each of the three policy domains. We then produced heatmaps for each state, documenting the number of policies present in each county (range 0–22). For these plots, policies were coded as binary (i.e., no policy versus any policy).

### Secondary analyses

We next conducted several secondary analyses to account for possible bias introduced by the imputation process. To do so, for each analysis above, we calculated the results using data obtained directly from counties only, without imputation using state data.

Second, to allow for greater variation and nuance in policy landscapes, we calculated an overall index of comprehensiveness, rather than simply the number of policies implemented. For this analysis, if there was no policy, this was coded as 0, the most comprehensive were coded as 1, and intermediate categories were fractions thereof. For example, for public events, no restriction was coded as 0, minimal (≥ 50% capacity) limitations was 0.25, major (< 50% capacity) limitations was 0.50, recommended cancellation was 0.75, and required cancellation was 1. When summing the policies to achieve a total policy score for each county, the range was again 0–22, with non-integer values possible.

Finally, we used principal component analysis (PCA) as an alternative technique to create a composite index of policy comprehensiveness (see Supplemental Methods for details). We also examined the contributing policies to each of the principal components created by this technique to assess the relationships between the different policies in a different manner than the pairwise correlations described above.

## Results

Overall, policy data were most readily available for states and urban and suburban counties. Rural county data collection was more challenging since policies often were not documented or implemented, or websites were not updated regularly. Policy information was most often derived from health department and other government websites.

### Variation in county-level COVID-19-related policies

Univariate analyses demonstrated substantial variation in the presence and comprehensiveness of county policies that were in effect during January-March 2021 (Table 2). For containment and closure policies (Panel A), the percent of counties with no restrictions in place for a given policy ranged from 0% for public events, to more than half for public transportation (67.8%), hair salons (52.6%), and religious gatherings (52.0%). For counties with restrictions, there were fewer restrictions in effect for schools, workplaces, restaurants, and childcare settings, with more restrictions for private gatherings and bars. Few policies demonstrated lack of documentation in this main analysis.

**Table 2:** Comprehensiveness of county-level policies, by domain (January-March 2021)

**Panel A. Containment and Closure Policies**	**No policy documented**	**No restrictions**	**1: Least comprehensive**	**2**	**3**	**4: Most comprehensive**
School closing	0.0%	1.2%	76.0%	15.2%	7.6%	-
Workplace closing	0.0%	5.3%	67.3%	8.2%	19.3%	-
Cancel public events	0.0%	0.0%	22.8%	28.7%	29.2%	19.3%
Restrictions on private gatherings	0.0%	0.6%	0.6%	3.5%	24.0%	71.3%
Close public transport	2.3%	67.8%	28.7%	1.2%	-	-
Stay at home requirements	0.0%	15.2%	64.9%	19.9%	-	-
Gym closing	0.0%	12.3%	55.0%	12.9%	19.9%	-
Restaurant closing	0.0%	3.5%	66.1%	5.3%	15.8%	9.4%
Bar closing	0.0%	4.1%	37.4%	9.4%	12.9%	36.3%
Movie theater closing	2.9%	3.5%	58.5%	9.4%	25.7%	-
Childcare closing	1.8%	4.1%	93.6%	0.6%	-	-
Hair salon/barber shop closing	0.0%	52.6%	29.8%	8.2%	9.4%	-
Restrictions on religious gatherings	0.0%	52.0%	17.0%	31.0%	-	-
Nursing home visitation restrictions	0.0%	7.0%	84.8%	8.2%	-	-
Curfew requirement	22.8%	34.5%	42.7%	-	-	-
**Panel B. Economic Response Polices**	**No policy documented**	**No support**	**Policy present**			
Housing financial support	0.0%	23.4%	76.6%	-	-	-
Utility support	12.3%	22.8%	64.9%	-	-	-
**Panel C. Public Health Policies**	**No policy documented**	**No policy**	**1: Least comprehensive**	**2**	**3**	**4: Most comprehensive**
Public information campaigns	0.0%	11.7%	17.5%	70.8%	-	-
Testing policy	0.0%	1.8%	1.2%	7.6%	89.5%	-
Contact tracing	1.8%	2.9%	33.9%	61.4%	-	-
Facial coverings	0.0%	0.6%	12.3%	20.5%	66.7%	-
Vaccination policy	4.7%	1.2%	7.0%	26.3%	56.7%	4.1%

Note: *N* = 171 counties in 7 states. Different policies have different numbers of possible categories, and dashes “-” indicate that a given category was not relevant or coded for a given policy. For counties with missing data on a given policy, data from state policies were used to infer local county policies

For economic response policies (Table 2, Panel B), 76.6% of counties had housing support, while 64.9% had utility relief. For public health policies (Table 2, Panel C), most were comprehensive, with 70.8% of county public information campaigns coordinated across traditional and social media, and 66.7% requiring facial coverings outside the home at all times. The percent of counties with no documentation for a given policy was less than 5%.

Results for analyses using county data only, without imputation using state data, are available in the Supplemental Results.

### Correlations between county-level COVID-19-related policies

We next examined correlations between each pair of policies (Table 3). Containment and closure policies were more likely to be correlated with each other, with nearly half (38.1%) of correlations positive and moderate (0.4–0.59) to strong (> 0.6) [23]. Most negative correlations were very weak (< 0.2). Meanwhile, correlations with and between economic response and public health policies included both positive and negative relationships, although these tended to be weak (0.2–0.39) or very weak, indicating less bundling for these policies. All correlations in this analysis were statistically significant at *p* < 0.05.

**Table 3:** Pairwise correlations between county-level COVID-19-related policies (January-March 2021)

	School closing	Workplace closing	Cancel public events	Restrictions on private gatherings	Close public transport	Stay at home requirements	Gym closing	Restaurant closing	Bar closing	Movie theater closing	Childcare closing
School closing	1.00										
Workplace closing	0.66	1.00									
Cancel public events	0.47	0.42	1.00								
Restrictions on private gatherings	-0.12	-0.23	0.17	1.00							
Close public transport	0.22	0.27	0.23	-0.07	1.00						
Stay at home requirements	0.49	0.59	0.42	-0.15	0.40	1.00					
Gym closing	0.66	0.70	0.46	-0.21	0.36	0.76	1.00				
Restaurant closing	0.67	0.73	0.61	-0.08	0.37	0.69	0.88	1.00			
Bar closing	0.43	0.56	0.28	-0.27	0.10	0.55	0.56	0.54	1.00		
Movie theater closing	0.67	0.67	0.65	-0.02	0.31	0.63	0.84	0.90	0.45	1.00	
Childcare closing	0.21	0.21	0.29	0.21	0.19	0.18	0.23	0.30	0.21	0.26	1.00
Hair salon/barber shop closing	0.30	0.29	0.19	0.24	0.29	0.33	0.40	0.39	0.08	0.34	0.25
Restrictions on religious gatherings	0.63	0.64	0.55	-0.03	0.28	0.61	0.80	0.79	0.44	0.83	0.25
Nursing home visitation restrictions	0.24	0.03	0.21	0.05	0.01	-0.00	0.13	0.17	0.11	0.14	0.19
Curfew requirement	0.19	0.18	0.29	0.21	0.20	0.31	0.32	0.34	0.10	0.28	0.23
Housing financial support	-0.06	-0.01	-0.10	0.11	0.17	0.02	-0.05	-0.10	-0.04	-0.17	-0.06
Utility support	0.06	-0.06	-0.00	-0.10	0.10	0.06	0.03	0.04	-0.08	-0.01	-0.16
Public information campaigns	0.32	0.26	0.37	0.03	0.09	0.30	0.40	0.43	0.20	0.51	0.23
Testing policy	-0.04	-0.23	-0.08	-0.00	-0.07	-0.16	-0.13	-0.16	-0.10	-0.11	0.08
Contact tracing	0.23	0.19	0.23	0.05	0.23	0.19	0.27	0.34	-0.02	0.41	0.05
Facial coverings	0.22	0.34	0.21	0.09	0.06	0.23	0.26	0.35	0.14	0.36	0.29
Vaccination policy	-0.09	-0.09	-0.09	-0.13	-0.14	0.07	0.06	-0.09	0.16	0.02	-0.16

	Hair salon/barber shop closing	Restrictions on religious gatherings	Nursing home visitation restrictions	Curfew requirement	Housing financial support	Utility support	Public information campaigns	Testing policy	Contact tracing	Facial coverings	Vaccination policy
School closing											
Workplace closing											
Cancel public events											
Restrictions on private gatherings											
Close public transport											
Stay at home requirements											
Gym closing											
Restaurant closing											
Bar closing											
Movie theater closing											
Childcare closing											
Hair salon/barber shop closing	1.00										
Restrictions on religious gatherings	0.53	1.00									
Nursing home visitation restrictions	0.14	0.13	1.00								
Curfew requirement	0.74	0.59	-0.06	1.00							
Housing financial support	0.16	-0.04	-0.01	0.14	1.00						
Utility support	-0.17	-0.15	0.13	-0.33	0.32	1.00					
Public information campaigns	0.09	0.30	0.02	-0.02	-0.22	-0.02	1.00				
Testing policy	-0.12	-0.10	-0.03	-0.09	-0.10	-0.01	-0.06	1.00			
Contact tracing	0.08	0.15	0.05	-0.12	0.04	0.20	0.27	0.07	1.00		
Facial coverings	0.06	0.20	0.06	-0.06	0.03	-0.01	0.24	-0.11	0.24	1.00	
Vaccination policy	-0.24	0.02	0.01	-0.17	-0.05	-0.01	0.10	0.09	-0.19	-0.05	1.00

Note: *N* = 171 counties in 7 states. Values in each cell represent Spearman’s correlation coefficients between each set of individual policies. For counties with missing data on a given policy, data from state policies were used to infer local county policies

### Geographic variation in county-level COVID-19-related policies

When comparing across states (Table 4), there was substantial variation in the mean number of COVID-19-related policies that counties in each state had in place. For containment and closure policies, this ranged from 5.5 policies passed per county in Mississippi to 14.1 in New York (out of 15 total possible). For economic response policies, this ranged from 1.1 in New York to 2.0 in Mississippi (out of 2 total possible). For public health policies, this ranged from 3.5 in Mississippi to 5.0 in California and New Jersey (out of 5 total possible).

**Table 4:** Mean number of county policies, by policy domain and state (January-March 2021)

*Policy domain (range)*	California	Louisiana	Mississippi	New Jersey	New York	Texas	Utah	Overall
Containment/closure (0–15)	13.4	13.9	5.5	14.0	14.1	10.6	10.5	12.1
Economic response (0–2)	1.5	1.2	2.0	1.3	1.1	1.4	1.6	1.4
Public health (0–5)	5.0	4.4	3.5	5.0	4.9	4.9	4.7	4.8
Total number of policies (0–22) ± SD	19.8 ± 1.7	19.5 ± 1.2	11.0 ± 3.4	20.3 ± 1.0	20.1 ± 1.5	16.9 ± 1.5	16.8 ± 2.2	18.2 ± 2.5
Number of counties	34	29	4	11	14	50	29	171

Note: *N* = 171 counties in 7 states. *SD* Standard deviation. For counties with missing data on a given policy, data from state policies were used to infer local county policies

**Fig. 1:**
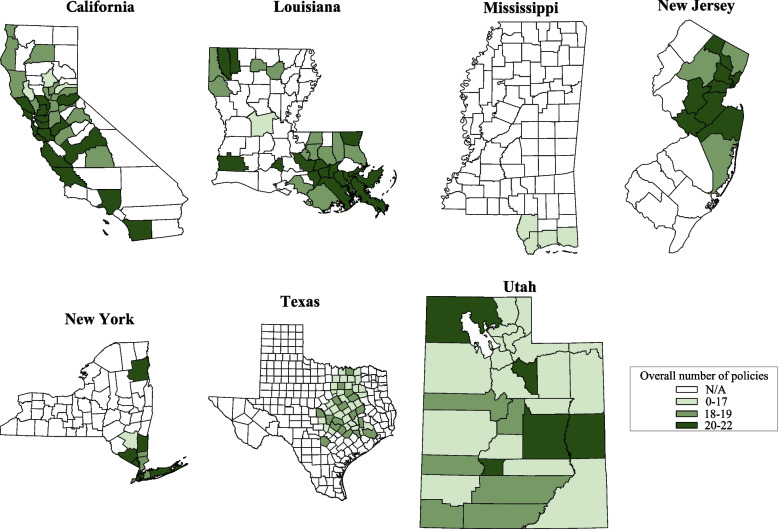
Distribution of Number of County Policies, by State (January-March 2021) Note: *N* = 171 counties in 7 states. Categories were created by splitting number of policies per county by tertile. For counties with missing data on a given policy, data from state policies were used to infer local county policies. This study focused on counties that corresponded to the places of residence for PCORnet and CCS patients, represented here in shades of green. Counties in white (“N/A”) were not included in the current study

Analyses also demonstrated variation in the number of county-level policies *within* states (Fig. 1; note that counties without shading were not included in our data collection). Most states had counties that fell into all three tertiles, with California, New York, and Utah having more counties in the top tertile. There were no counties with zero policies implemented.

When we examined scores for comprehensiveness of policies instead of summing binary indicators for whether any type of policy had been implemented (Supplemental Fig. 2), the variation across states again became even more pronounced. For example, counties in California were more likely to be in the top tertile, and counties in Louisiana were more likely to fall into lower tertiles.

### PCA results

We found that the first four principal components produced by the PCA loaded on combinations of policies that could be described as follows: (1) entity closures (e.g., business closures), (2) individual restrictions (e.g., on private gatherings), (3) state-funded programs (e.g., housing support), and (4) public health measures (e.g., vaccination). These were similar to the relationships we observed in the pairwise correlations described above. See Supplemental Methods and Supplemental Table 7 for additional details.

PCA results also indicated that counties in different states varied in the values of each of the different principal components (Supplemental Table 8). For example, counties in California and New York had the highest mean scores for the first principal component (entity closure). Meanwhile, counties in Texas and Mississippi had the highest mean scores on the second principal component (individual restrictions) followed by the counties in Mississippi, while New Jersey and Utah had the highest scores on the third principal component (state funding). Counties in New York and Texas had the highest scores on the fourth principal component (public health measures). The composite score shows that, overall, comprehensiveness of policies was greatest in counties in New York and California. Each principal component and the composite policy index also demonstrated substantial variation within states (Supplemental Fig. 4).

## Discussion

Assembling data from a variety of government and media websites, the U.S. COVID-19 County Policy (UCCP) Database is among the first to provide granular data on local governments’ policy responses to the COVID-19 pandemic. In the current study, we present findings from the first phase of data collection, including data from 171 counties across 7 states in the U.S. We document variation across and within states on 22 types of containment, economic, and public health policies, finding that there were important geographic differences in the types and comprehensiveness of policies to address the COVID-19 pandemic’s health and economic consequences. This adds to a body of literature that has examined variation in COVID-19 policymaking globally across countries and domestically at the state level [11, 24, 25], and suggests that future research examining the impact of policies on health during the pandemic needs to consider local variation that may result in substantial heterogeneity within states.

Several factors may explain the differences in county-level policymaking. These include health-related factors (like COVID-19 case rates, vaccines, or underlying morbidity and age distributions that may spur county policymakers to act), or sociodemographic characteristics (like population density or urbanicity that may have heightened concern for transmission). Local economic factors—e.g., unemployment or poverty rates—may have also driven policymaking around economic support for local residents. Alternatively, a stronger history of public health policymaking—e.g., around HIV/AIDS in the San Francisco Bay Area—may have led to more active COVID-19-related policymaking. Prior work has examined state characteristics associated with public health policymaking to address obesity [26], and future work could similarly examine the predictors of COVID-19-related policymaking.

It is also likely that the variation in policymaking in this study resulted in county variation in health outcomes. Studies before COVID-19 have often demonstrated the impacts of public health and economic policies on health and social outcomes [27–29], and a handful of studies during the COVID-19 pandemic have addressed similar questions, e.g., finding that state shelter-in-place policies affected mobility [30]. This is an important area of future research, to inform policymaking during the later phases of this pandemic and future crises. Notably, research on the health impacts of COVID-19-related policies will be complicated by the collinearity of some policies (demonstrated here) and confounding by other unobserved place-based factors.

While patterns of policy comprehensiveness were similar when using county-only versus imputed county-plus-state data, the number of policies per county in each state varied much more. Specifically, some states were more likely to have policies enacted at the state level rather than by counties. There are several possible explanations for these findings. During this phase of the pandemic, these counties—which tend to be located in more rural states—may have been experiencing more limited transmission relative to counties in more urban states, such that county policymakers felt less urgency to act. Alternately, these counties are also located in states that are more right-leaning on indicators of political conservativeness [21], and which have been previously documented to have a less forceful policy response to COVID-19 in part perhaps due to a political philosophy supporting a more limited role of government [24]. Relatedly, these states are more likely to engage in preemption of local policies, in which state governments restrict county governments’ abilities to act on a given issue [31]. We did not capture state pre-emption policies, but pre-emption is known to affect other types of health policies like tobacco control and firearm safety [31]. Future research can examine the extent to which this may have played a role in constraining county COVID-19-related policies. Interestingly, these same states also tend to be bigger proponents of federalism, in which authority over local affairs is ceded to states rather than the federal government, including for public health issues [32]. It appears that the same logic does not extend to counties being afforded more flexibility when managing their own local affairs, despite the proliferation of local public health agencies in recent decades [32].

This study has several strengths, including the collection of policy data spanning 171 diverse counties in 7 states. We collected 22 policies across numerous domains, painting a detailed portrait of the policy landscape during this period of active policymaking and COVID-19 transmission. This study also has limitations. Counties were selected for inclusion based on the criteria of the parent project (see Supplement), and therefore do not constitute a generalizable or representative sample nor cover all 50 states. Similarly, data were collected in January-March 2021, and do not capture earlier or later phases of the pandemic. Nevertheless, even in this sample that is limited in geographic and temporal scope, we demonstrate substantial variation, and provide evidence that such data collection endeavors of county policies are critical to understanding how the pandemic has unfolded. Moreover, in ongoing work, we are conducting retrospective longitudinal weekly data collection for the period 2020–2021 from a larger swath of U.S. counties in all 50 states and Washington D.C. to fill this gap, and the current database will be updated with new data as it becomes available. Additionally, while the initial data collection presented here involved 22 policies hypothesized to be important for health, in ongoing work we are collecting additional data on a more robust set of social and economic support policies that may be important for addressing health disparities. Finally, policies are not always implemented or enforced as intended; importantly, we did not intend to capture actual enforcement or on-the-ground conditions, but rather the policies that were passed by governments during this time.

## Conclusion

This study provides among the first evidence of variation in county-level COVID-19-related policies across multiple states, demonstrating substantial differences that reflect an active and diverse policymaking landscape. Assembling such data is the first step in developing evidence on how these policies contributed to and may continue to influence pandemic-related health and socioeconomic outcomes and disparities therein, and this study thereby informs future research on the health impacts of these policies. We encourage other investigators to leverage these data, which we are making publicly available, to advance research in this realm.

## Supplementary Information

**Additional file 1.**

## Data Availability

The datasets used for the current study are available to any investigator at openICPSR, a service of the Inter-university Consortium for Political and Social Research: 10.3886/E180482V1.

## Abbreviation

PCA: Principal component analysis
UCCP: U.S. COVID-19 County Policy
