# Supplementary material for: The U.S. COVID-19 County Policy Database: a novel resource to support pandemic-related research
Source: BMC Public Health. 2022 Oct 10;22:1882. doi: 10.1186/s12889-022-14132-6 (PMC9548418; doi:10.1186/s12889-022-14132-6)
Supplement: Supplementary file 1 — Additional file 1. [file 12889_2022_14132_MOESM1_ESM.pdf]

SUPPLEMENTAL METHODS

County Selection

The goal of the parent project for this study was to link county policy data to health outcomes derived from health systems participating in the National Patient Centered Outcomes Research Network (PCORnet) Common Data Model[1] and patient-reported outcomes from the Eureka COVID-19 Citizen Science study (CCS).[2] Thus, while there are over 3,000 counties in the U.S., this first wave of data collection for the U.S. COVID-19 County Policy (UCCP) Database focused on counties that corresponded to the places of residence for PCORnet and CCS patients. Data collection for the UCCP Database is ongoing and it will eventually include additional time points for 2020–2021 and additional counties nationwide in all 50 states and Washington, D.C.

While public health policymaking may occur at other geographic levels, e.g., cities; local public health departments in the U.S. are almost always situated within county governments, which is why we focused data collection at this level. There are a few exceptions, e.g., in Massachusetts, public health initiatives occur at the municipality level; note that Massachusetts was not included in this sample. In cases where we were unable to locate information for a given policy at the county level, we also checked whether the largest city within that county had implemented any relevant policies (as long as the city's population made up at least 51% of the county's population).

Data Collection

For each policy, data collectors abstracted the scoring, the date of data collection, and source documentation. This included the source's URL as well as a PDF of the source to capture information in real time given the potential for website content to change. These sources are

available in the online data repository for this paper at openICPSR (see main manuscript for link to data repository).

Prior to the start of data collection, research staff received training and were assigned a subset of counties on which to gather policy data. The study team developed documentation to serve as a guide for data collectors. This documentation provided detailed guidance for each policy to enhance consistency in coding of nuances encountered. For example, if public events were banned but an exception was granted for one large event with social distancing, the public events policy indicator would be scored as "require cancelling." The training documentation also reflected general guidance on how to handle instances in which source materials on government websites were outdated or undated, and instances in which a county deferred to or referenced state policy. A central file was maintained through which data collectors posed questions, for adjudication by the study leads. Training materials were updated regularly to reflect additional guidance related to adjudication questions. The lead data collector also conducted weekly random checks of a subset of data collection points to provide feedback to individual data collectors and to review in team meetings. For the longitudinal data collection that is currently ongoing for the UCCP Database, we are carrying out double data entry of 10% of entries to evaluate inter-rater reliability.

Note that future investigators may choose to rank the comprehensiveness of categories differently than how we did so for the present study. For example, for public events, we decided that minimal (≥50% capacity) limitations and major (<50% capacity) limitations were less comprehensive than a recommended cancellation, since there was still an implication that events could proceed as scheduled. However, future researchers may decide that the capacity limitations were more restrictive because they had enforceable limits rather than representing simply a

"recommendation." Interested researchers can therefore re-order these categories in the raw data that we provide in the online data repository.

Principal Components Analysis

PCA is commonly used as an unsupervised machine learning technique to reduce the dimensionality of large data sets. In essence, it creates multiple sets of weighted averages of each of the policies, producing principal components that explain the most variance in the data. Because the policy indicators are categorical, we specifically used polychoric PCA to account for the discrete and ordinal data structure, as standard PCA is intended for use with continuous variables.[3, 4]

The scree plot in Supplemental Figure 3 shows that after the fourth component, eigenvalues dropped dramatically, and the additional information added by the next component was very small. We therefore used four principal components as they explained 79% of the variation in policies. Supplemental Tables 6 and 7 show the first four components of the orthogonally rotated principal components and which factors they loaded, respectively. As seen in Supplemental Table 6, the first principal component explained 35% of the total variance, the second 20%, the third 16%, and the fourth 8%. According to Supplemental Table 7, the first principal component mostly loaded on variables that define entity closures such as closings of schools, workplaces, bars, and religious places. The second principal component mostly loaded on restrictions for individuals, such as restrictions to private gatherings, nursing homes, and curfew requirements. The third component mostly loaded on policies that required state funding, such as housing and utility support, public information campaigns, and contact tracing. Finally, the fourth component mostly loaded on policies related to public health measures such as testing and vaccination policies.

Finally, we created a composite index by adding all four of the principal components to measure the degree of policy comprehensiveness. Geographic distributions of the four individual principal components and the composite index that sums these four components are included in Supplemental Figures 4a-4e.

SUPPLEMENTAL RESULTS

Analyses Without Imputation Using State Data

For containment and closure policies, when using data from county sources only, without imputation of missing values using state data, patterns were similar to the main analysis, but with more missingness (Supplemental Table 3, Panel A). For economic response policies, values for housing support and utility relief were 34.5% and 26.9%, respectively, indicating that economic response policies were primarily implemented by states (Supplemental Table 3, Panel B). for public health policies, 40.9% were missing policy documentation for contact tracing and 17.5% for testing and facial coverings, again suggesting that states were more active than counties in implementing these policies (Supplemental Table 3, Panel C).

When examining cross-state geographic variation in county-level COVID-19-related policies (Supplemental Table 5), there was more variation than in the main analysis, with fewer policies passed in Louisiana, Mississippi, and Utah. This may be due in part to the fact that these states had higher levels of missingness at the county level (Supplemental Table 4). For example, using these unimputed data, the number of economic response policies for each of these states was 0.1, 0.0, and 0.1, respectively. This indicates that there was more policymaking happening at the state level in these locations.

When examining geographic variation in county policies within states (Supplemental Figure 1), the variation across states again became more pronounced. California, New Jersey, and New York had a greater number of policies across counties, with most in the top tertile. Meanwhile, Louisiana, Mississippi, Texas, and Utah had more counties in the bottom tertile. As above, this suggests the relative importance of state-level policymaking in some states. Only the county of Falls, Texas, had no policy restrictions or policy documentation of any kind.

SUPPLEMENTAL REFERENCES

1. Forrest CB, McTigue KM, Hernandez AF, Cohen LW, Cruz H, Haynes K, Kaushal R, Kho AN, Marsolo KA, Nair VP *et al*: **PCORnet® 2020: current state, accomplishments, and future directions**. *Journal of clinical epidemiology* 2021, **129**:60-67.
2. Beatty AL, Peyser ND, Butcher XE, Carton TW, Olgin JE, Pletcher MJ, Marcus GM: **The COVID-19 Citizen Science Study: Protocol for a Longitudinal Digital Health Cohort Study**. *JMIR Research Protocols* 2021, **10**(8):e28169.
3. Martel P, Mbofana F, Cousens S: **The polychoric dual-component wealth index as an alternative to the DHS index: Addressing the urban bias**. *Journal of Global Health* 2021, **11**.
4. Kolenikov S, Angeles G: **Socioeconomic status measurement with discrete proxy variables: Is principal component analysis a reliable answer?** *Review of Income and Wealth* 2009, **55**(1):128-165.

Supplemental Table 1. List of Counties for Which Policy Data Were Collected, by State

California	Louisiana	Mississippi	New Jersey	New York	Texas	Utah
Alameda	Ascension	Hancock	Bergen	Bronx	Austin	Beaver
Amador	Assumption	Harrison	Essex	Dutchess	Bastrop	Box Elder
Butte	Bossier	Jackson	Hudson	Essex	Bell	Cache
Contra Costa	Caddo	Pearl River	Mercer	Kings	Bexar	Carbon
Del Norte	Calcasieu		Middlesex	Nassau	Blanco	Daggett
El Dorado	De Soto		Monmouth	New York	Bosque	Davis
Fresno	East Baton Rouge		Morris	Orange	Brazos	Duchesne
Humboldt	East Feliciana		Ocean	Putnam	Burleson	Emery
Lake	Iberville		Passaic	Queens	Burnet	Garfield
Los Angeles	Jefferson		Somerset	Richmond	Collin	Grand
Marin	Lafayette		Union	Rockland	Cooke	Iron
Mendocino	Lafourche			Suffolk	Coryell	Juab
Merced	Lincoln			Ulster	Dallas	Kane
Monterey	Livingston			Westchester	Denton	Millard
Napa	Orleans				Ellis	Morgan
Nevada	Ouachita				Falls	Piute
Placer	Plaquemines				Fannin	Rich
Sacramento	Rapides				Fayette	Salt Lake
San Diego	St. Bernard				Freestone	San Juan
San Francisco	St. Charles				Grayson	Sanpete
San Joaquin	St. James				Grimes	Sevier
San Luis Obispo	St. John the Baptist				Hamilton	Summit
San Mateo	St. Mary				Harris	Tooele
Santa Clara	St. Tammany				Hays	Uintah
Santa Cruz	Tangipahoa				Henderson	Utah
Shasta	Terrebonne				Hill	Wasatch
Solano	Washington				Hood	Washington
Sonoma	Webster				Hunt	Wayne
Stanislaus	West Baton Rouge				Johnson	Weber
Sutter					Kaufman	
Tulare					Lampasas	
Tuolumne					Lee	
Yolo					Leon	
Yuba					Limestone	
					Llano	
					McLennan	
					Milam	
					Montgomery	
					Navarro	
					Parker	
					Robertson	
					Rockwall	

(continued on next page)

	San Saba
	Tarrant
	Travis
	Van Zandt
	Waller
	Washington
	Williamson
	Wise

Note: Counties are not intended to be a representative sample of U.S. counties, but rather were selected for the purposes of linkage with health outcomes from the National Patient Centered Outcomes Research Network (PCORnet) Common Data Model and patient-reported outcomes from the Eureka COVID Citizen Science Study (CCS). The total population of these counties is over 90.4 million individuals.

Supplemental Table 2. Scoring Criteria for Policies Included in the Study

**Policy**	*Least comprehensive*	→			*Most comprehensive*	
Panel A. Containment and Closure Policies						
School Closing	0 - No measures (i.e., no restrictions)	1 - Recommend closing or all schools open with alterations resulting in significant differences compared to non-COVID-19 operations	2 - Require closing (only some levels or categories - e.g., just high school)	3 - Require closing all levels		
Workplace Closing	0 - No measures (i.e., no restrictions)	1 - Recommend closing (or recommend work from home)	2 - Require closing (or work from home) for some sectors or categories of non-essential office workers	3 - Require closing (or work from home) all-but-essential workplaces (e.g., grocery stores, doctors, pharmacies)		
Cancel Public Events	0 - No measures (i.e., no restrictions)	1 - Events allowed, with minimal (>=50% capacity) limitations	2 - Events allowed, with major (<50% capacity) limitations	3 - Recommend cancelling	4 - Require cancelling	
Restrictions on Private Gatherings	0 - No restrictions	1 - Restrictions on very large gatherings (the limit is above 1000 people)	2 - Restrictions on gatherings between 101-1000 people	3 - Restrictions on gatherings between 11-100 people	4 - Restrictions on gatherings of 10 people or less	
Close Public Transport	0 - No measures (i.e., no restrictions)	1 - Recommend closing (or significantly reduce volume/route/means of transport available)	2 - Require closing (or prohibit most from using it)			
Stay at Home Requirements	0 - No measures (i.e., no restrictions)	1 - Recommend not leaving house	2 - Require not leaving house with exceptions for daily exercise, grocery	3 - Require not leaving house with minimal exceptions (e.g., allowed to leave only once a		

			shopping, and 'essential' trips	week, or only one person can leave at a time)		
Gym Closing	0 - No measures (i.e., no restrictions)	1 - Open, with minimal (>=50% capacity) limitations	2 - Open, with major (<50% capacity) limitations	3 - Closed		
Restaurant Closing	0 - No measures (i.e., no restrictions)	1 - Open for indoor dining, with minimal (>=50% capacity) limitations	2 - Open for indoor dining, with major (<50% capacity) limitations	3 - Outdoor only (with or without takeout/delivery)	4 - Takeout/delivery only	5 - Closed
Bar Closing	0 - No measures (i.e., no restrictions)	1 - Open for indoor drinking, with minimal (>=50% capacity) limitations	2 - Open for indoor drinking, with major (<50% capacity) limitations	3 - Outdoor only (with or without takeout/delivery)	4 - Takeout/delivery only	5 - Closed
Movie Theater Closing	0 - No measures (i.e., no restrictions)	1 - Open, with minimal (>=50% capacity) limitations	2 - Open, with major (<50% capacity) limitations	3 - Closed		
Childcare Closing	0 - No measures (i.e., no restrictions)	1 - Recommend closing or all day cares open with alterations resulting in significant differences compared to non-COVID-19 operations	2 - Require closing (only some levels or categories - e.g., babies)	3 - Require closing all levels		
Hair Salon/Barber Shop Closing	0 - No measures (i.e., no restrictions)	1 - Open, with minimal (>=50% capacity) limitations	2 - Open, with major (<50% capacity) limitations	3 - Closed		
Restrictions on Religious Gatherings	0 - No measures (i.e., no restrictions)	1 - Open, with minimal (>=50% capacity) limitations	2 - Open, with major (<50% capacity) limitations	3 - Closed		
Nursing Home Visitation Restrictions	0 - No measures (i.e., no restrictions)	1 - Visitation with limitations	2 - Visitation ban			
Curfew Requirement	0 - No measures (i.e., no restrictions)	1 - Required				

Panel B. Economic Support Policies						
Housing Financial Support	0 - No measures (i.e., no support)	1 - Support				
Utility Support	0 - No measures (i.e., no support)	1 - Support				
Panel C. Public Health Policies						
Public Information Campaigns	0 - No COVID-19 public information campaign	1 - Public officials urging caution about COVID-19	2 - Coordinated public information campaign (e.g., across traditional and social media)			
Testing Policy	0 - No testing policy	1 - Only those who both (a) have symptoms AND (b) meet specific criteria (e.g., essential workers, admitted to hospital, came into contact with a known case, returned from overseas)	2 - Testing of anyone showing COVID-19 symptoms	3 - Open public testing (e.g., "drive-through" testing available to asymptomatic people)		
Contact Tracing	0 - No contact tracing	1 - Limited contact tracing (not done for all cases)	2 - Comprehensive contact tracing (done for all identified cases)			
Facial Coverings	0 - No policy	1 - Recommended	2 - Required in **some** specified shared/public spaces outside the home with other people present, or **some** situations when social distancing not possible	3 - Required in **all** shared/public spaces outside the home with other people present or **all** situations when social distancing not possible	4 - Required outside the home at all times regardless of location or presence of other people	
Vaccination Policy	0 - No availability	1 - Availability for **ONE** of following:	2 - Availability for **TWO** of following:	3 - Availability for **ALL** of following:	4 - Availability for all three plus partial	5 - Universal availability

		essential workers/ clinically or socially vulnerable groups / elderly groups	essential workers/ clinically or socially vulnerable groups / elderly groups	essential workers/ clinically or socially vulnerable groups / elderly groups	additional availability (select broad groups/ages)	

Note: For each policy, there was also a category to indicate that there is no information available to determine policy in place.

Supplemental Table 3. Comprehensiveness of County-level Policies, by Domain, No Imputation Using State Data (January-March 2021)

Panel A. Containment and Closure Policies	No policy documented	No restrictions	1: Least comprehensive	2	3	4: Most comprehensive
School closing	4.7%	1.2%	71.3%	15.2%	7.6%	-
Workplace closing	24.0%	1.8%	52.0%	2.9%	19.3%	-
Cancel public events	26.3%	0.0%	15.8%	16.4%	22.2%	19.3%
Restrictions on private gatherings	29.8%	0.6%	0.6%	0.6%	24.0%	44.4%
Close public transport	41.5%	36.3%	21.1%	1.2%	-	-
Stay at home requirements	31.6%	7.0%	41.5%	19.9%	-	-
Gym closing	23.4%	3.5%	42.7%	10.5%	19.9%	-
Restaurant closing	21.1%	0.6%	48.5%	4.7%	15.8%	9.4%
Bar closing	18.1%	1.2%	27.5%	4.1%	12.9%	36.3%
Movie theater closing	25.7%	0.0%	42.7%	5.8%	25.7%	-
Day care closing	38.6%	4.1%	56.7%	0.6%	-	-
Hair salon/barber shop closing	25.1%	43.3%	17.5%	4.7%	9.4%	-
Restrictions on religious gatherings	22.8%	35.7%	11.7%	29.8%	-	-
Nursing home visitation restrictions	51.5%	2.9%	41.5%	4.1%	-	-
Curfew requirement	49.1%	17.5%	33.3%	-	-	-
**Panel B. Economic Response Polices**	**No policy documented**	**No support**	**Policy present**			
Housing financial support	42.7%	22.8%	34.5%	-	-	-
Utility support	50.3%	22.8%	26.9%	-	-	-
**Public Health Policies**	**No policy documented**	**No policy**	**1: Least comprehensive**	**2**	**3**	**4: Most comprehensive**
Public information campaigns	0.6%	11.7%	17.5%	70.2%	-	-
Testing policy	17.5%	1.8%	1.2%	6.4%	73.1%	-
Contact tracing	40.9%	2.9%	19.3%	36.8%	-	-
Facial coverings	17.5%	0.6%	11.1%	15.8%	55.0%	-
Vaccination policy	8.2%	1.2%	5.8%	24.0%	56.7%	4.1%

Note: N = 171 counties in 7 states. Different policies have different numbers of possible categories, and dashes "-" indicate that a given category was not relevant or coded for a given policy. For counties with missing data on a given policy, data from state policies was not used to infer local county policies.

Supplemental Table 4. Percent Missing Values Across All County Policies, by State and Rural/Urban Status

State	*Percent Missing*	
	Urban	Rural
California	6.7	13.6
Louisiana	54.7	22.7
Mississippi	63.6	77.3
New Jersey	20.7	0.0
New York	0.7	0.0
Texas	15.8	22.5
Utah	37.9	65.2
Overall	23.8	39.9

Note: N = 171 counties in 7 states. Data were collected for 22 county-level policies, and the values above represent percent missingness across all policies.

Supplemental Table 5. Mean Number of County Policies, by Policy Domain and State, No Imputation Using State Data (January-March 2021)

Policy domain (range)	CA	LA	MS	NJ	NY	TX	UT	Overall
Containment/closure (0-15)	13.1	7.2	3.3	10.0	13.9	9.1	4.5	9.1
Economic response (0-2)	0.8	0.1	0.0	1.2	1.1	0.8	0.1	0.6
Public health (0-5)	4.6	2.3	2.8	4.9	4.9	4.5	3.3	4.0
Total number of policies (0-22) ± SD	18.5 ± 2.2	9.7 ± 6.7	6.0 ± 5.4	16.1 ± 3.6	20.0 ± 1.6	14.4 ± 3.4	7.9 ± 5.4	13.7 ± 6.0
Number of counties	34	29	4	11	14	50	29	171

Note: N = 171 counties in 7 states. SD = standard deviation. For counties with missing data on a given policy, data from state policies were used to infer local county policies.

Supplemental Table 6. Summary of principal components

Component	*Variance*	*Proportion of variance*	*Cumulative proportion*
Component 1	7.60	0.35	0.35
Component 2	4.41	0.20	0.55
Component 3	3.58	0.16	0.71
Component 4	1.85	0.08	0.79

Supplemental Table 7. Loadings of rotated components

Variable	*Component 1*	*Component 2*	*Component 3*	*Component 4*
School closing	0.37	-	-	-
Workplace closing	0.37	-	-	-
Cancel public events	-	-	-	-
Restrictions on private gatherings	-	0.48	-	-
Close public transport	-	-	-	-
Stay at home requirements	-	-	-	-
Gym closing	0.33	-	-	-
Restaurant closing	-	-	-	-
Bar closing	0.36	-	-	-
Movie theatre closing	-	-	-	-
Childcare closing	-	-	-	-
Hair salon/barber shop closing	-	0.33	-	-
Restrictions on religious gatherings	0.36	-	-	-
Nursing home visitation restrictions	-	0.43	-	-
Curfew requirement	-	0.46	-	-
Housing financial support	-	-	0.47	-
Utility support	-	-	0.58	-
Public information campaigns	-	-	0.35	-
Testing policy	-	-	-	0.39
Contact tracing	-	-	0.35	-
Facial coverings	-	-	-	-
Vaccination policy	-	-	-	0.65

Note: Blank spaces represent absolute loadings that are less than 0.3.

Supplemental Table 8. Summary statistics of PCA-derived indices by state

	California		Louisiana		Mississippi		New Jersey		New York		Texas		Utah	
Component	Mean	SD	Mean	SD	Mean	SD	Mean	SD	Mean	SD	Mean	SD	Mean	SD
Entity closure index (PC1)	10.08	-1.04	3.27	2.49	1.74	1.93	5.83	1.54	8.11	0.98	4.90	1.40	2.50	1.86
Individual restriction index (PC2)	0.31	0.92	0.32	0.89	2.03	0.14	1.95	0.90	1.44	0.49	2.41	0.81	1.75	0.88
State funding index (PC3)	-3.75	1.22	-1.61	0.85	-1.45	0.32	-1.23	0.74	-0.88	0.50	-2.04	1.17	-1.02	0.60
Public health index (PC4)	1.68	0.58	1.30	1.29	1.89	1.14	1.55	0.85	2.63	0.72	2.13	0.99	1.16	1.15
Composite index	8.32	1.87	3.29	3.07	4.20	3.31	8.10	1.72	11.30	1.08	7.40	2.94	4.39	3.02

Note: Higher levels indicate stronger or more comprehensive policies for a given index.
PC = principal component; PCA = principal component analysis; SD = standard deviation.

Supplemental Figure 1. Distribution of Number of County Policies by State, No Imputation Using State Data (January-March 2021)

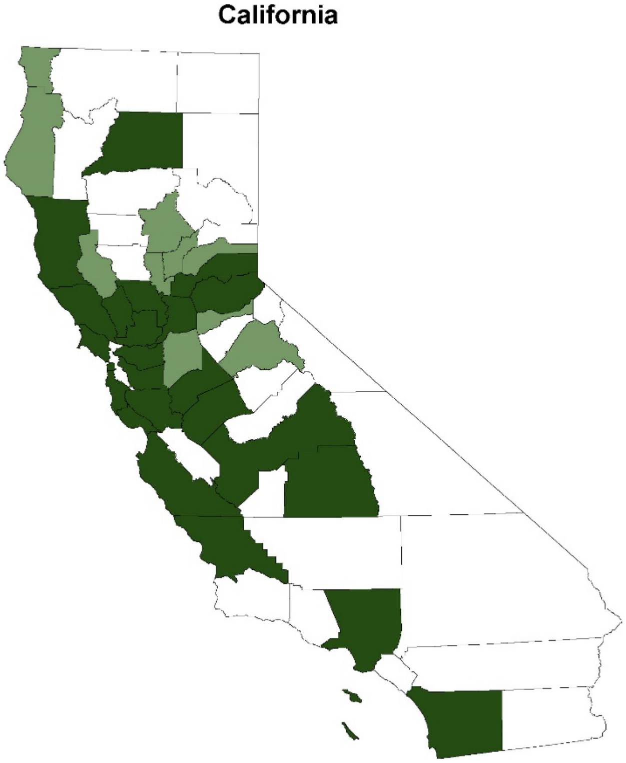

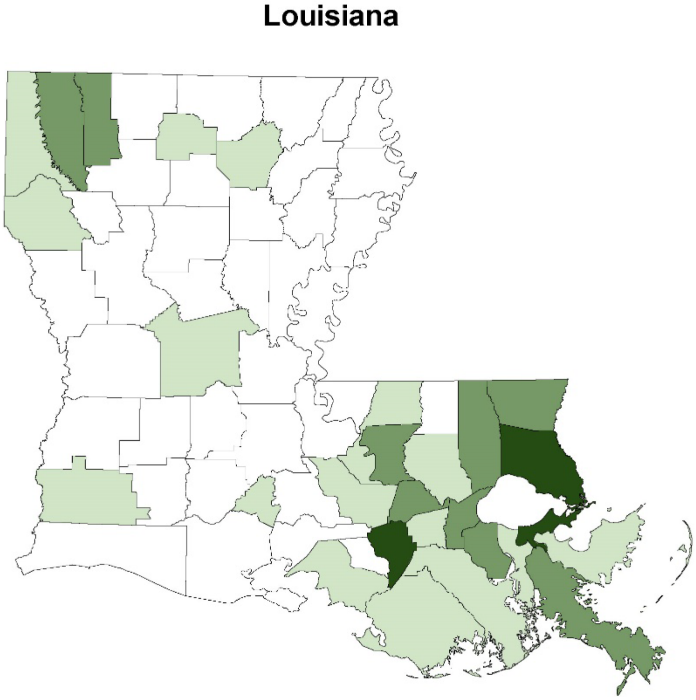

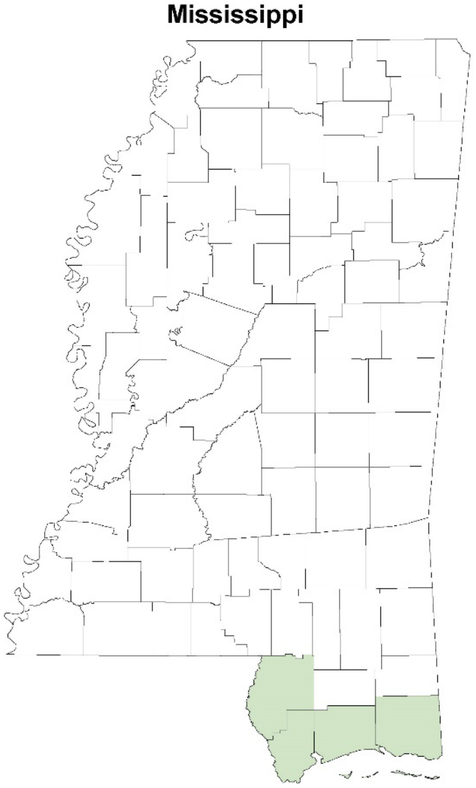

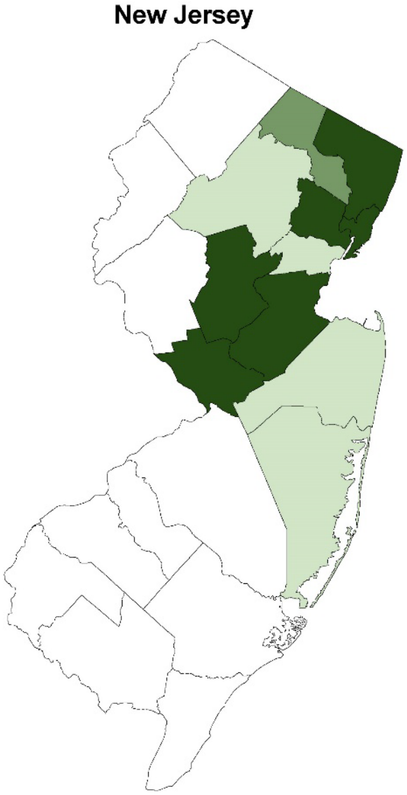

(continued on next page)

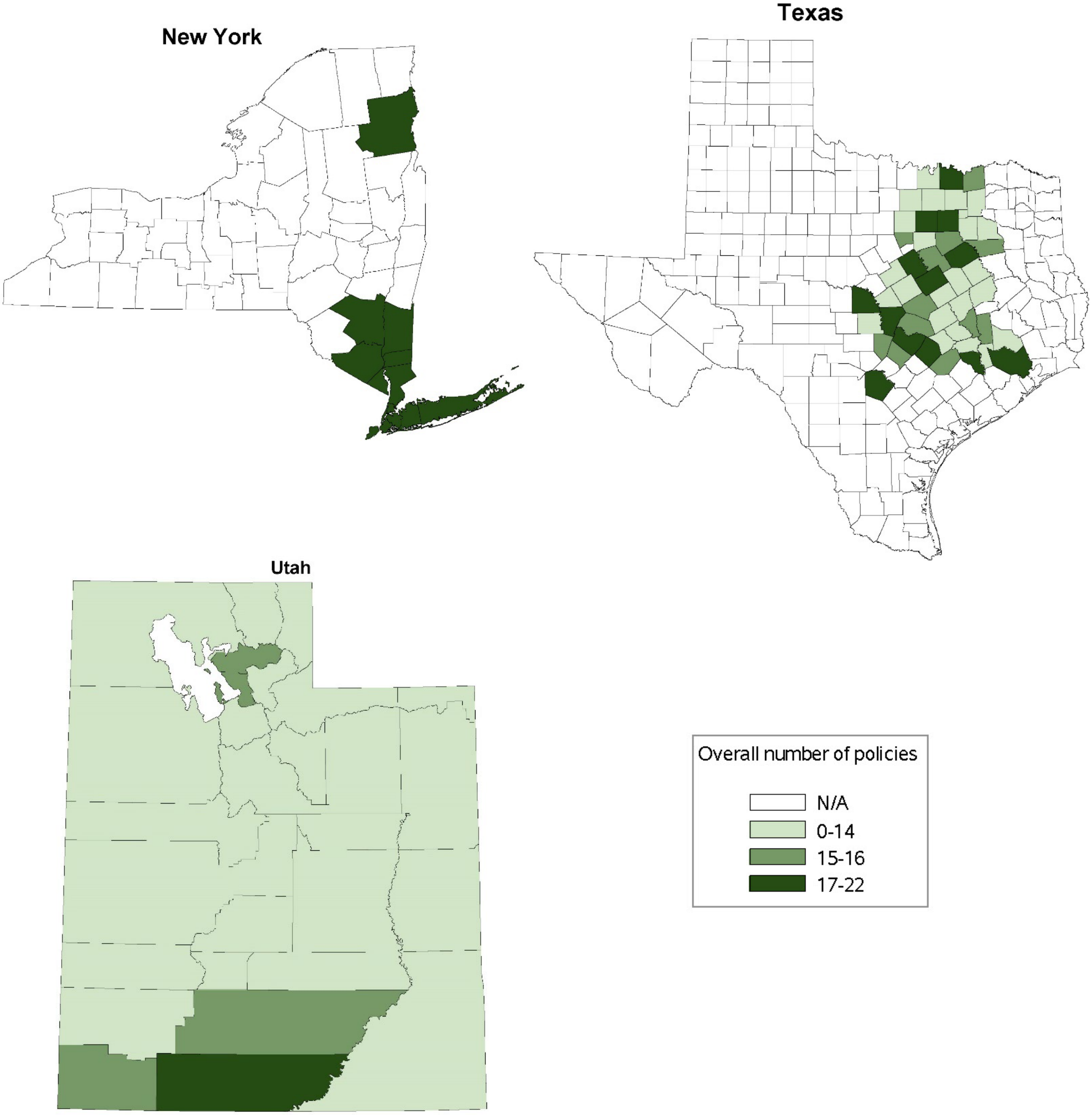

Note: N = 171 counties in 7 states. Categories were created by splitting number of policies per county by tertile. For counties with missing data on a given policy, data from state policies were not used to infer local county policies. This study focused on counties that corresponded to the places of residence for PCORnet and CCS patients, represented here in shades of green. Counties in white ("N/A") were not included in the current study.

Supplemental Figure 2. Distribution of Number of County Policies by State, Weighted by Policy Comprehensiveness (January-March 2021)

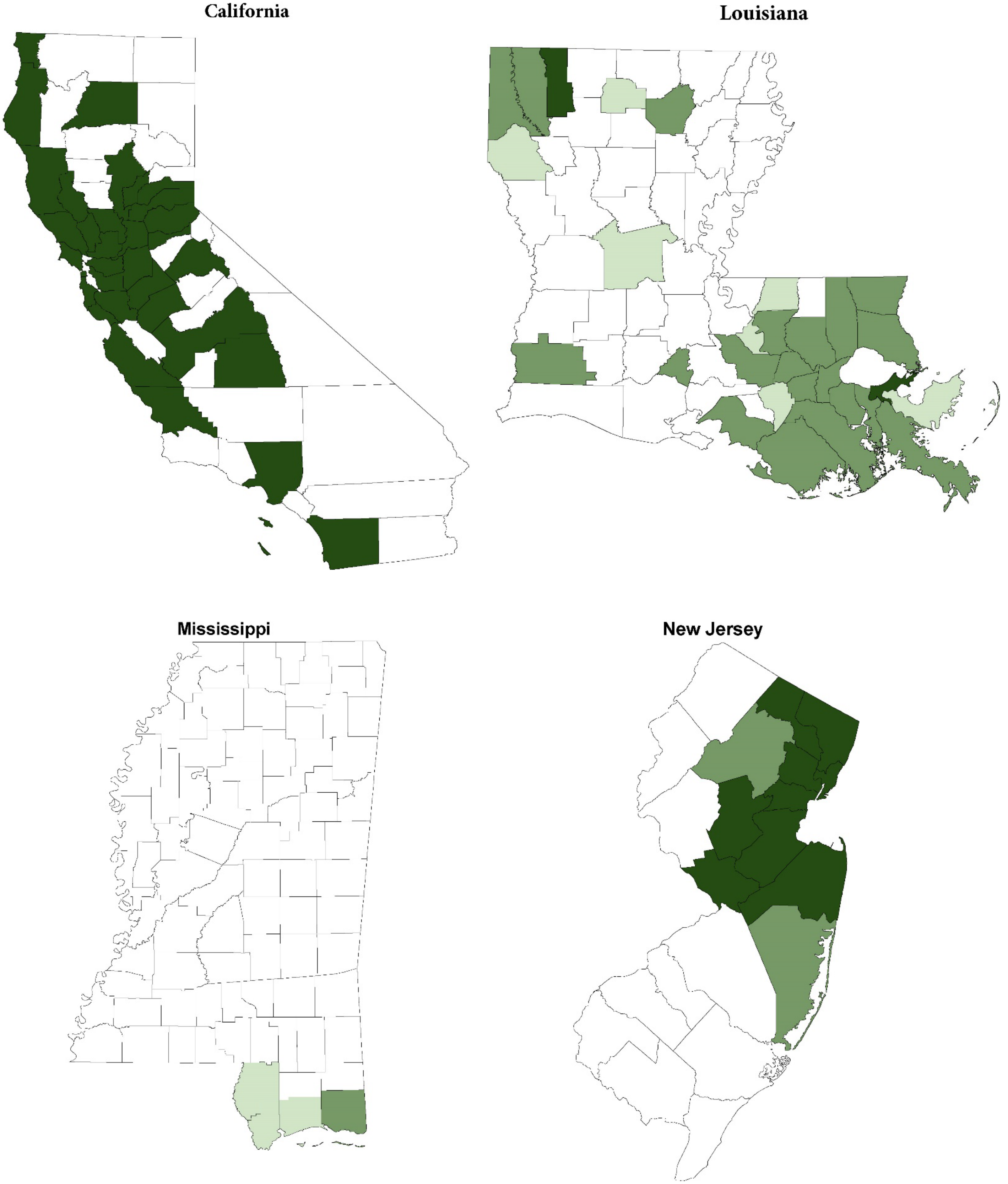

(continued on next page)

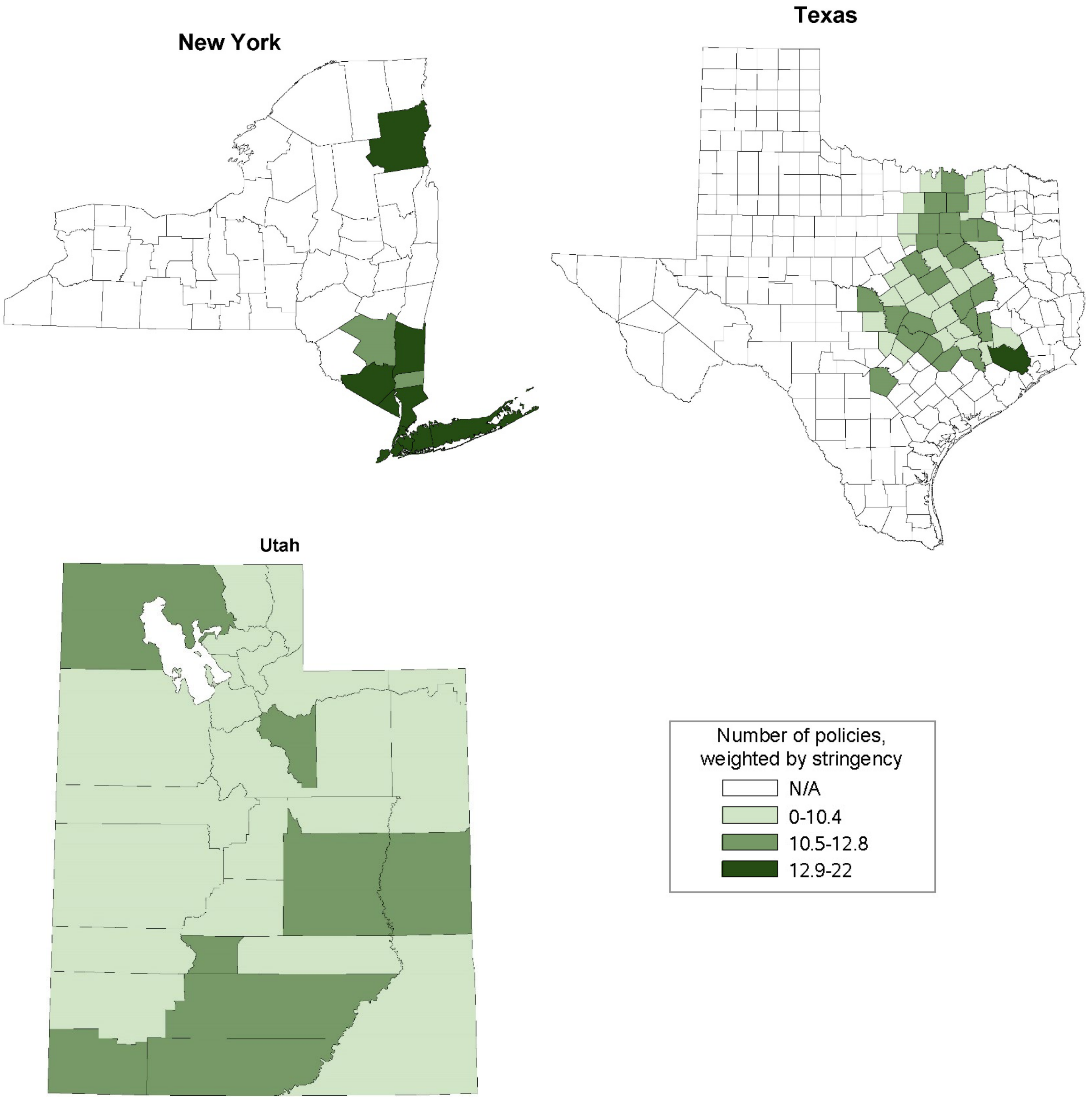

Note: N = 171 counties in 7 states. Categories were created by splitting number of policies per county by tertile. For counties with missing data on a given policy, data from state policies were used to infer local county policies. This study focused on counties that corresponded to the places of residence for PCORnet and CCS patients, represented here in shades of green. Counties in white ("N/A") were not included in the current study.

Supplemental Figure 3. Scree Plot of Eigenvalues After Polychoric Principal Component Analysis

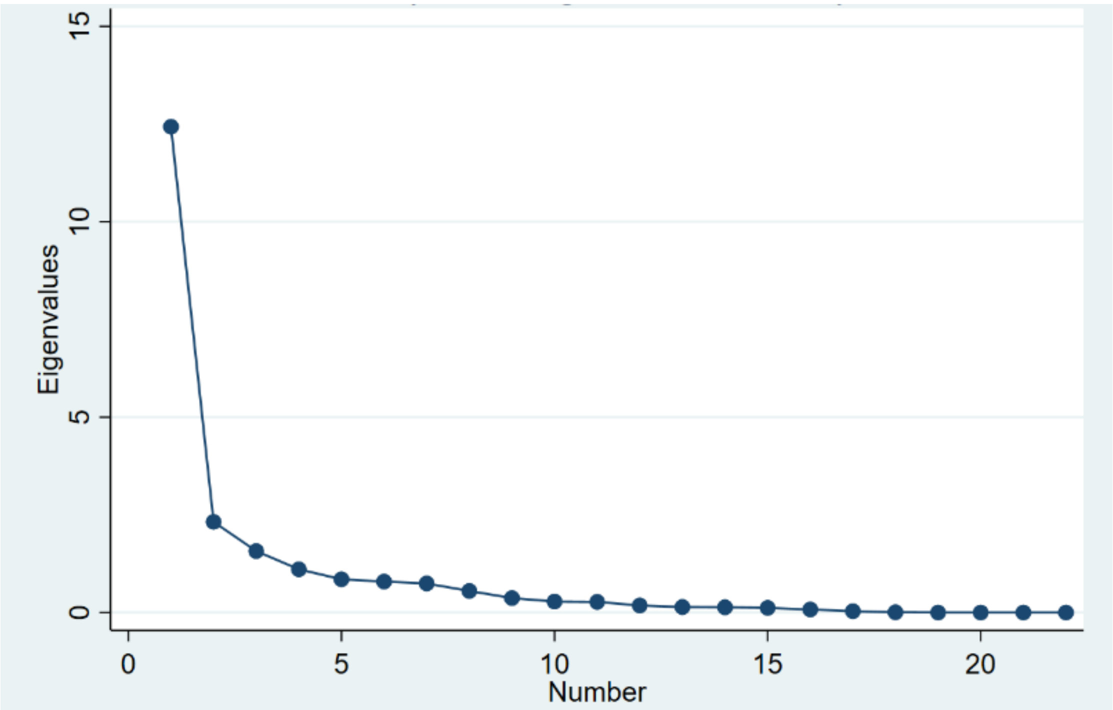

Supplemental Figure 4a. Distribution of the First Principal Component (Entity Closure)

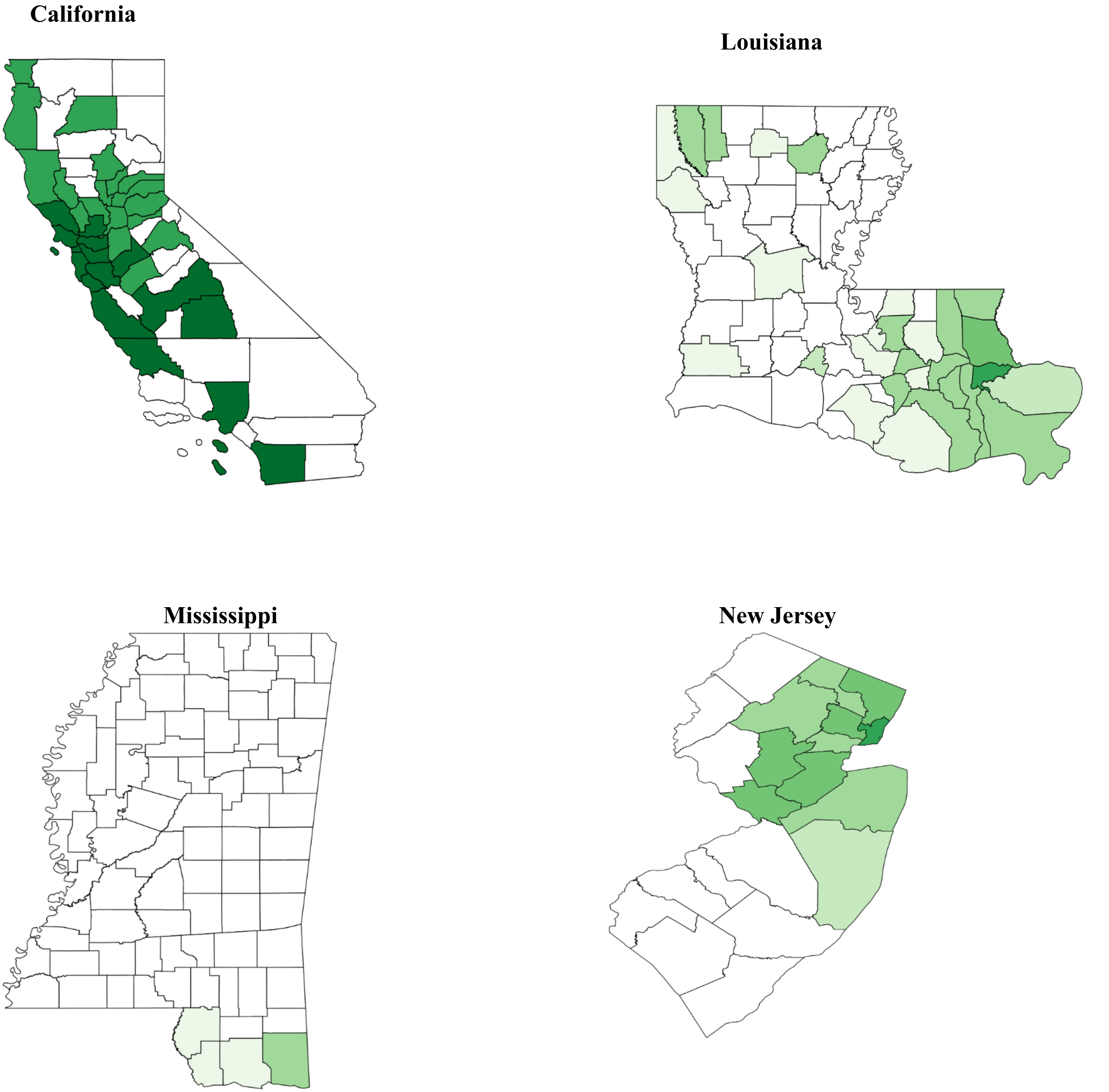

(continued on next page)

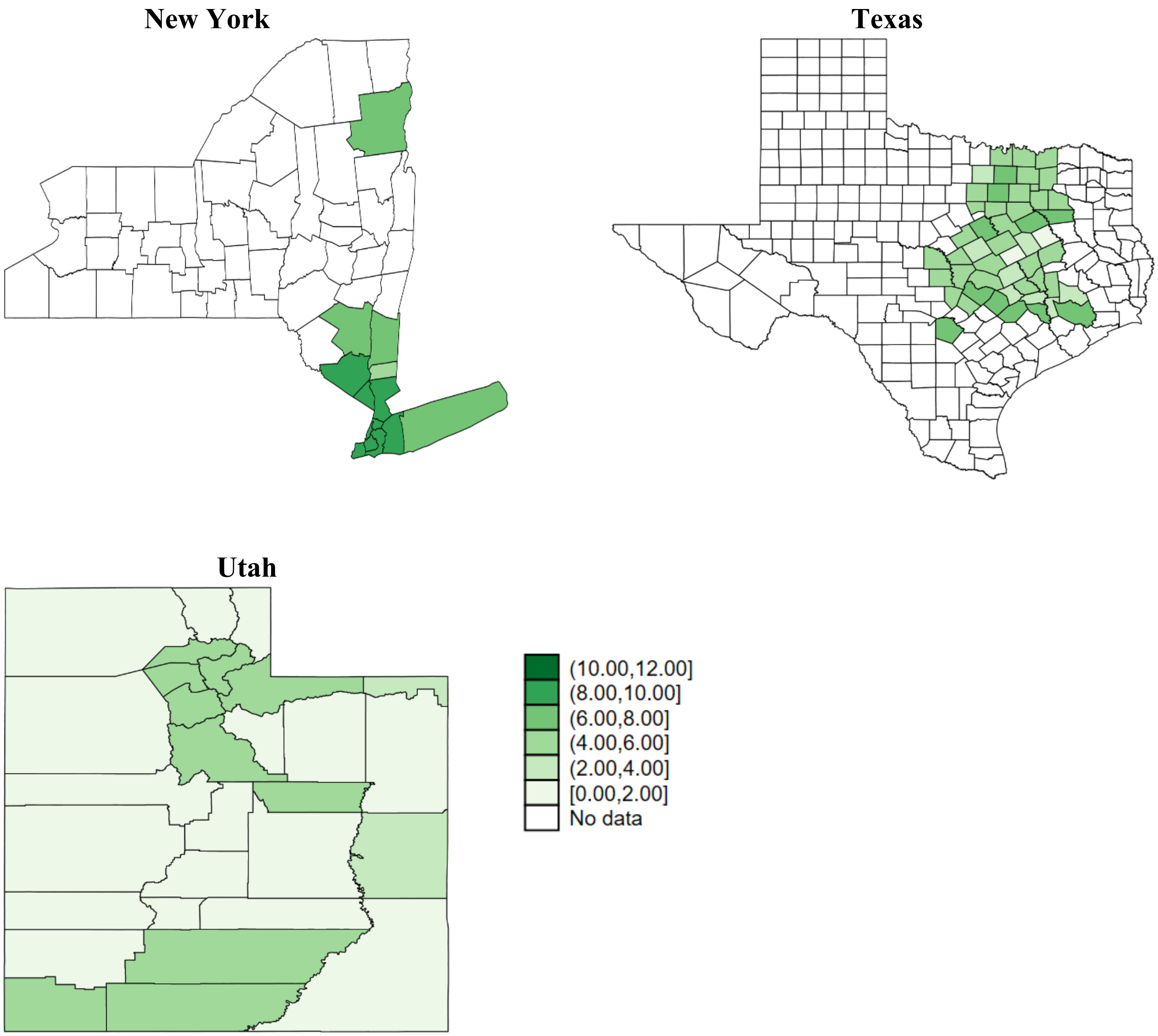
New York
Texas
Utah
(10.00,12.00]
(8.00,10.00]
(6.00,8.00]
(4.00,6.00]
(2.00,4.00]
[0.00,2.00]
No data

Supplemental Figure 4b. Distribution of the Second Principal Component (Individual Restrictions)

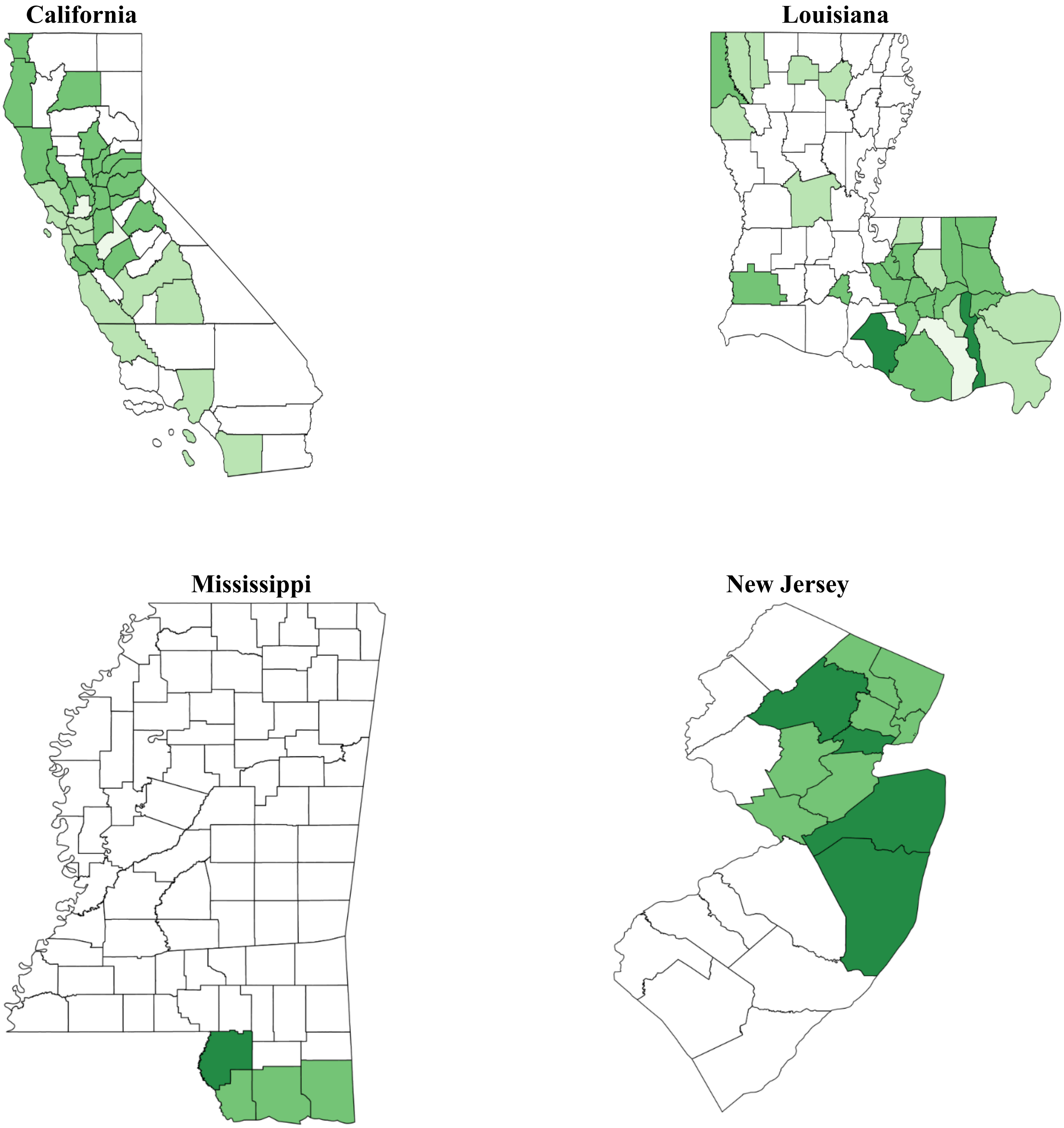

(continued on next page)

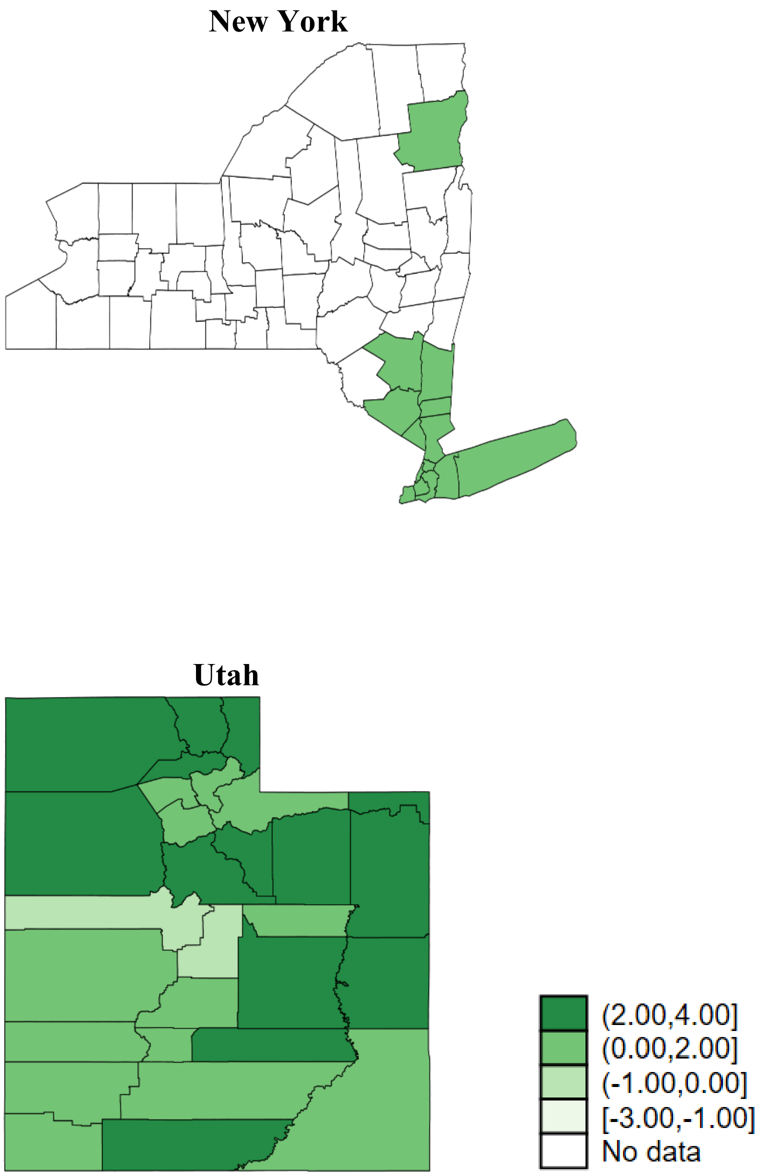
New York
Texas
Utah
(2.00,4.00]
(0.00,2.00]
(-1.00,0.00]
[-3.00,-1.00]
No data

Supplemental Figure 4c. Distribution of the Third Principal Component (State Funding)

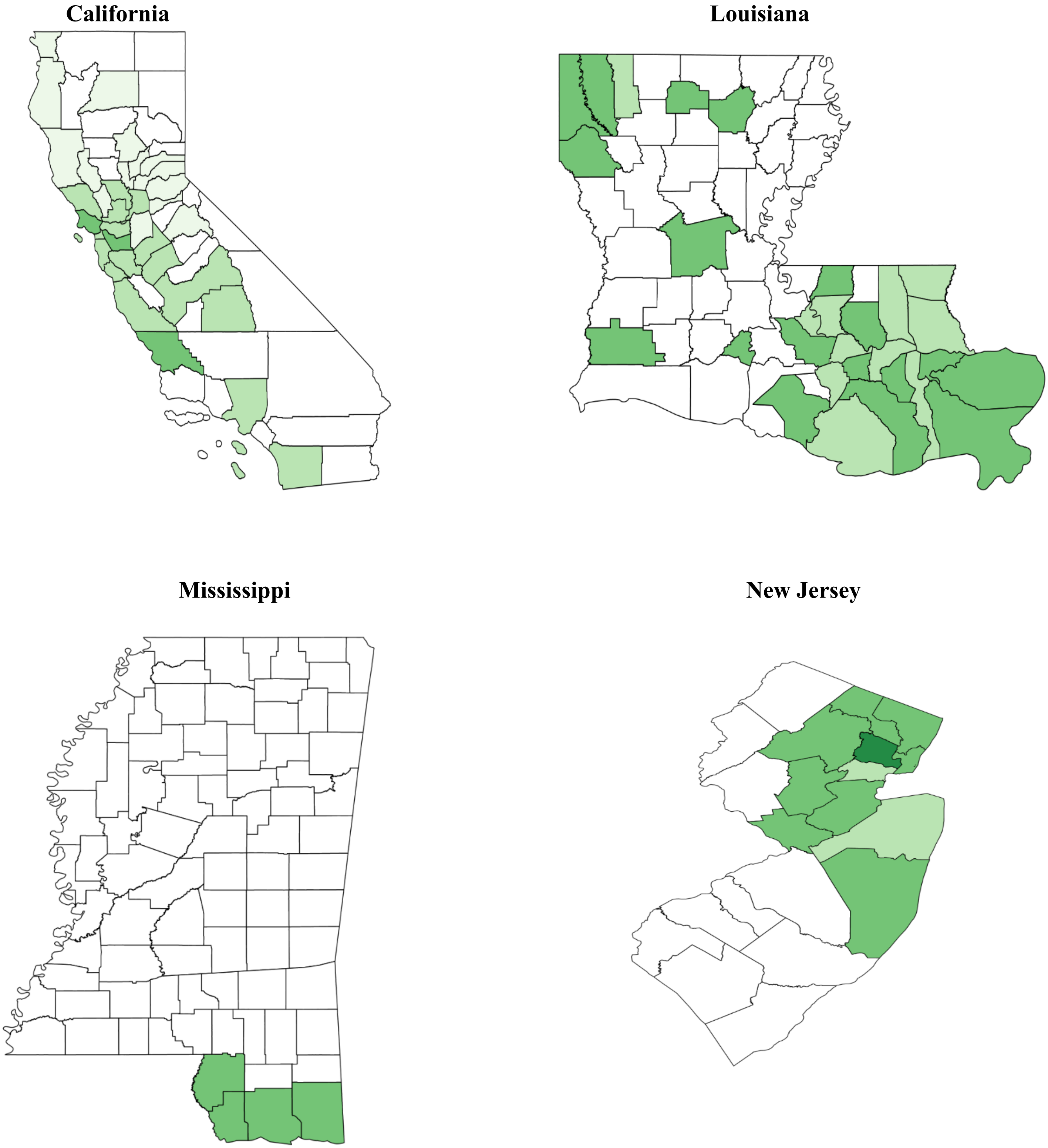

(continued on next page)

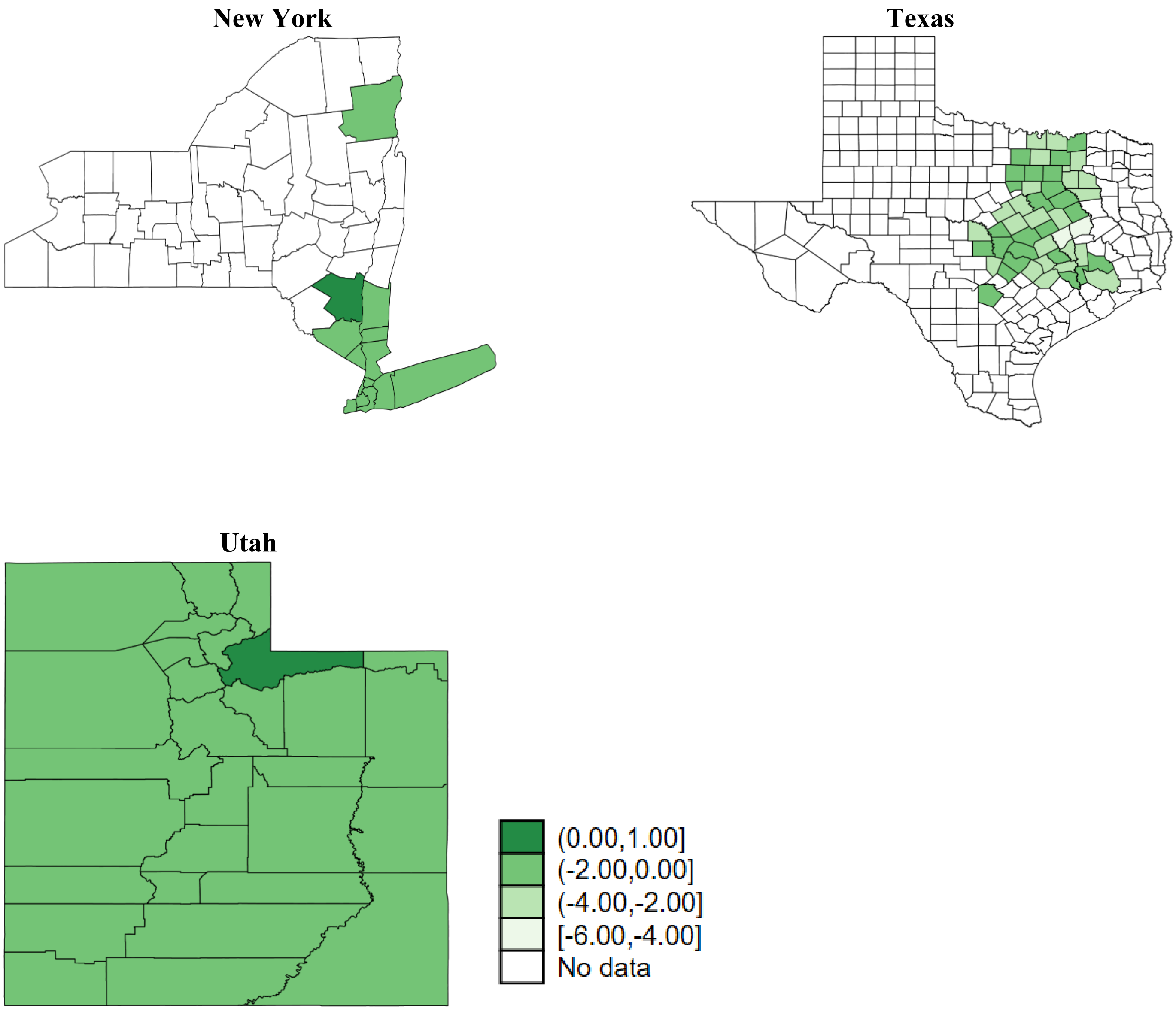
New York
Texas
Utah
(0.00,1.00]
(-2.00,0.00]
(-4.00,-2.00]
[-6.00,-4.00]
No data

Supplemental Figure 4d. Distribution of the Fourth Principal Component (Public Health Measures)

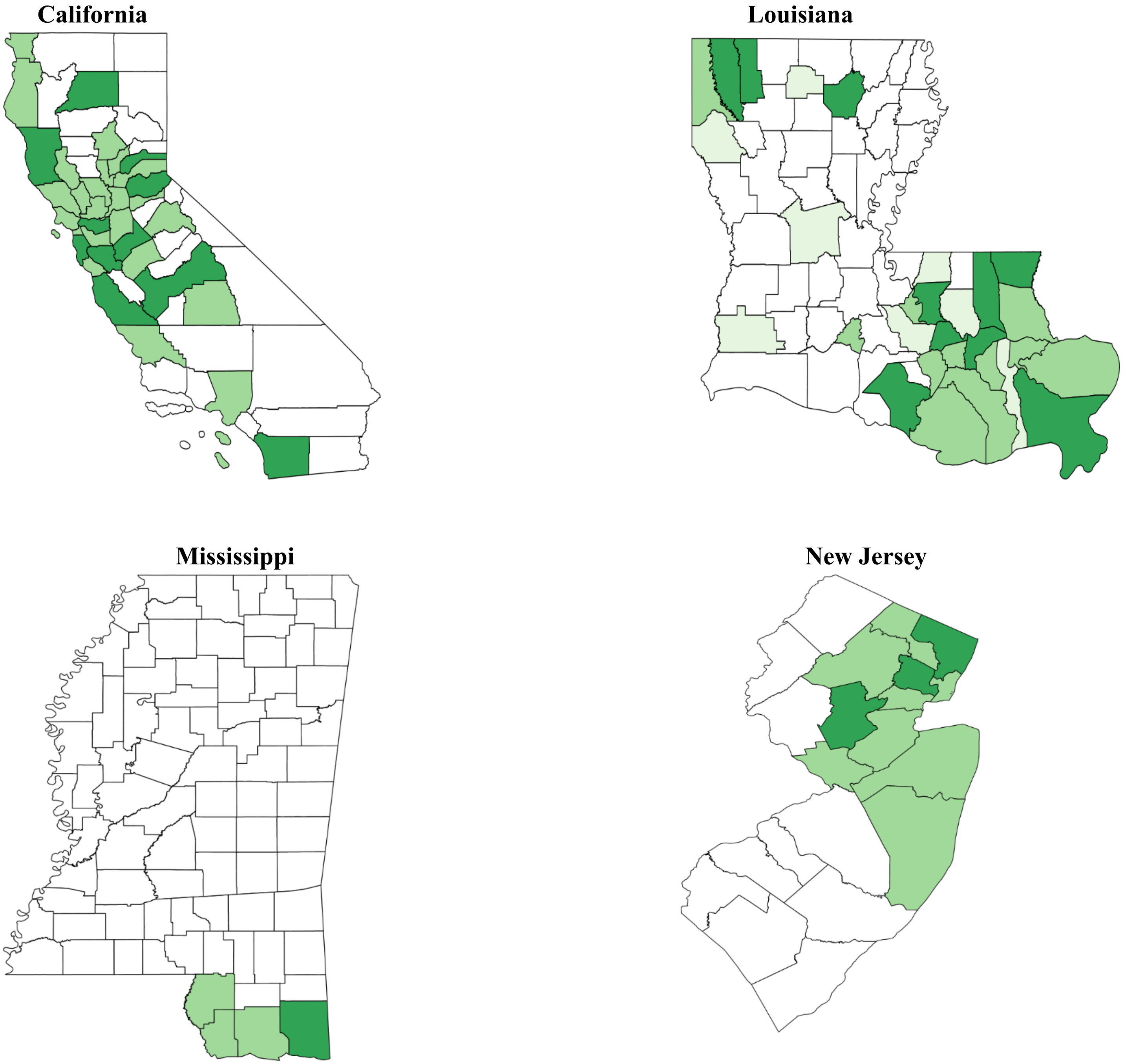

(continued on next page)

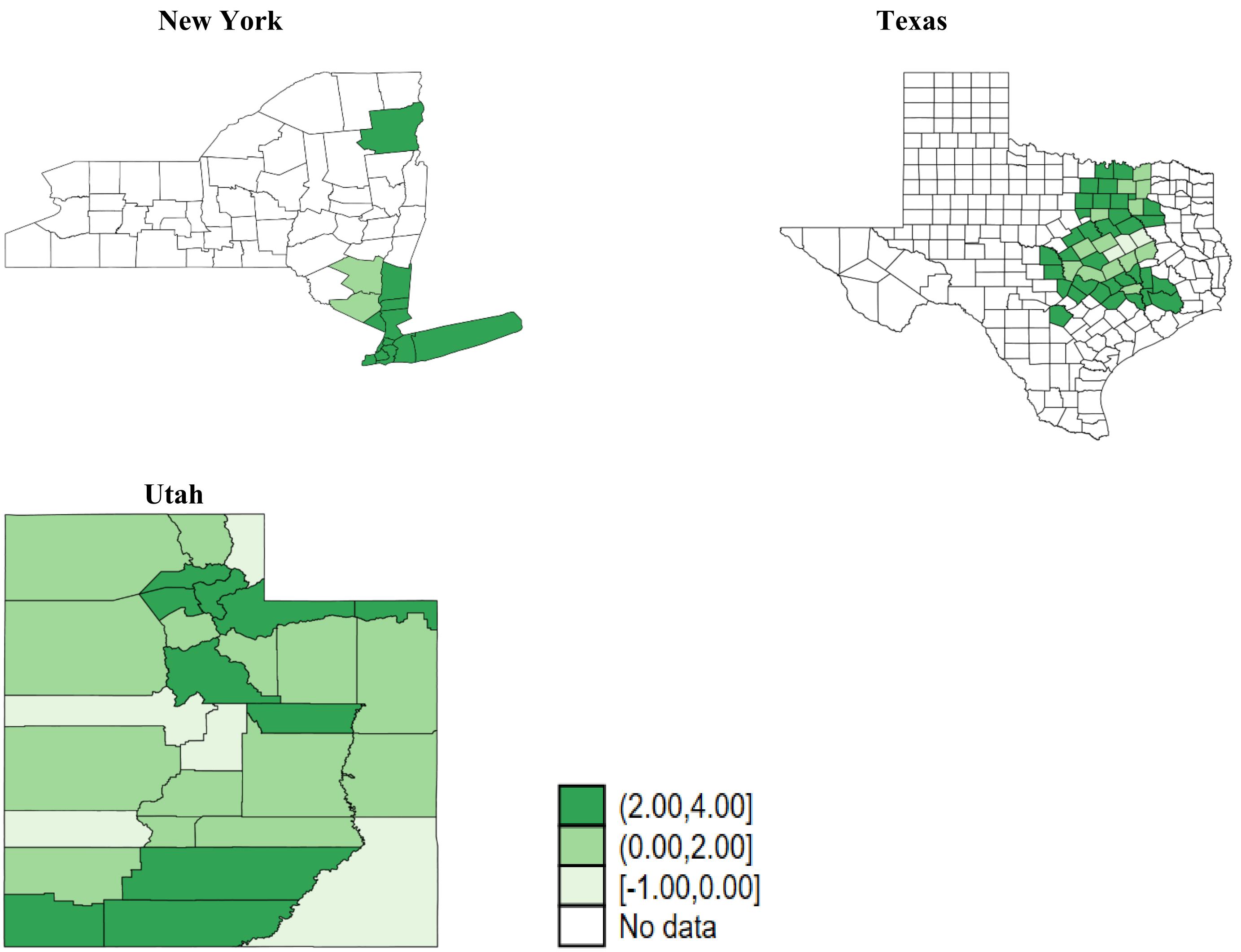
New York
Texas
Utah
(2.00,4.00]
(0.00,2.00]
[-1.00,0.00]
No data

Supplemental Figure 4e. Distribution of Composite Policy Index from Principal Component Analysis

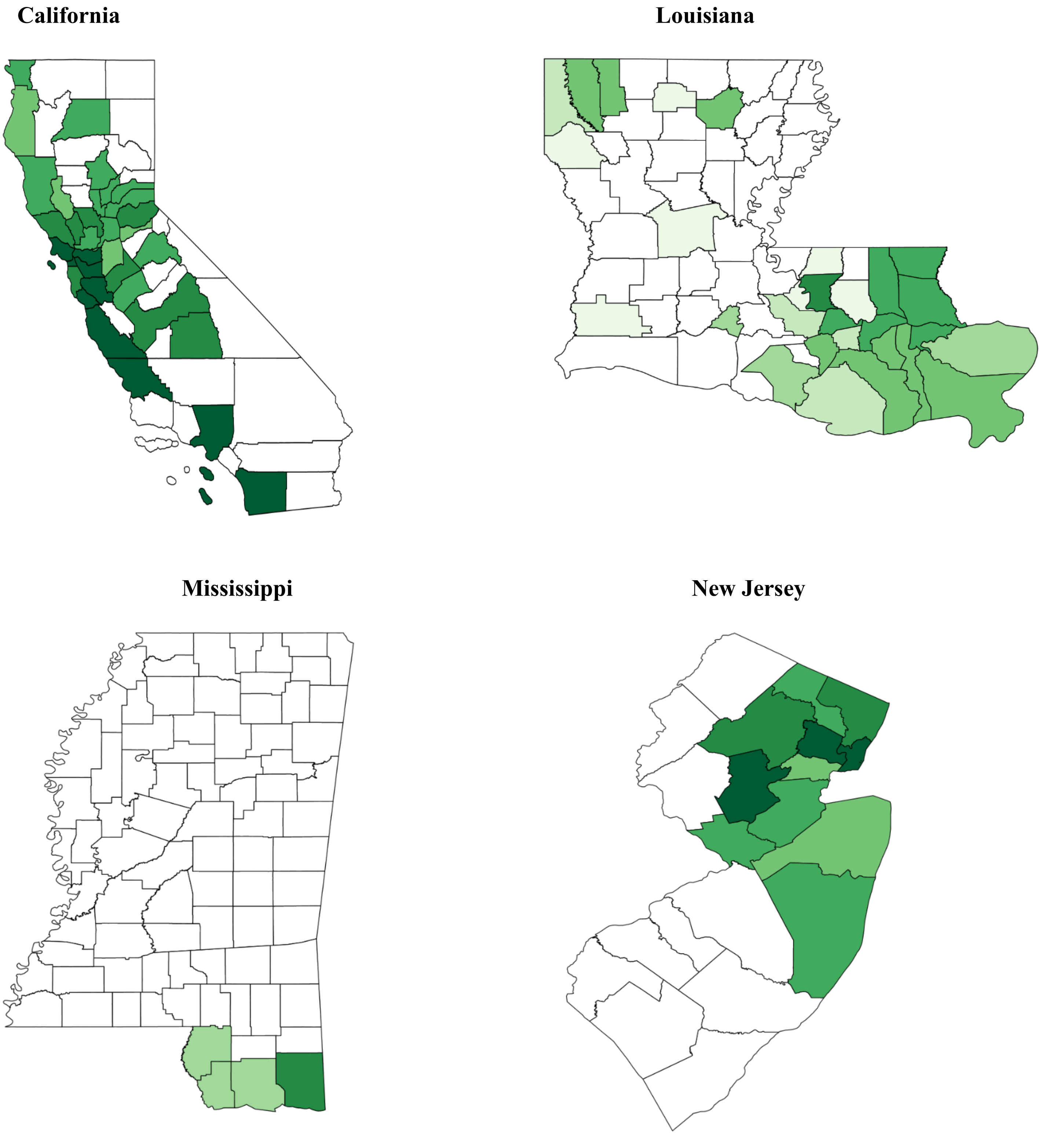

(continued on next page)

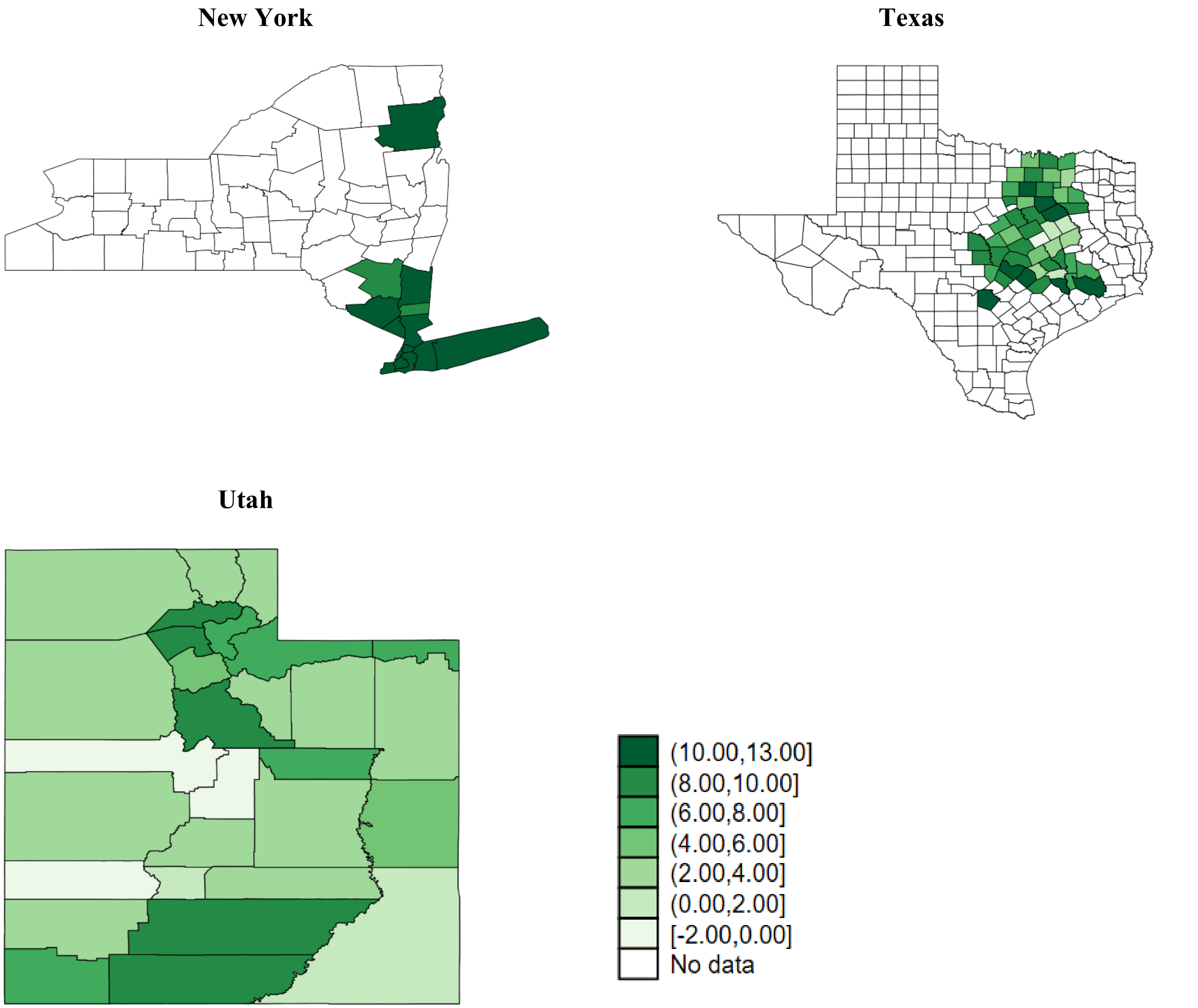
New York
Texas
Utah
(10.00,13.00]
(8.00,10.00]
(6.00,8.00]
(4.00,6.00]
(2.00,4.00]
(0.00,2.00]
[-2.00,0.00]
No data
